# Colivelin, a synthetic derivative of humanin, ameliorates endothelial injury and glycocalyx shedding after sepsis in mice

**DOI:** 10.3389/fimmu.2022.984298

**Published:** 2022-09-02

**Authors:** Catherine Urban, Hannah V. Hayes, Giovanna Piraino, Vivian Wolfe, Patrick Lahni, Michael O’Connor, Ciara Phares, Basilia Zingarelli

**Affiliations:** ^1^ Division of Pediatric Critical Care, Stony Brook Children’s, Stony Brook University, Stony Brook, NY, United States; ^2^ Department of Surgery, College of Medicine, University of Cincinnati, Cincinnati, OH, United States; ^3^ Division of Critical Care Medicine, Cincinnati Children’s Hospital Medical Center, Cincinnati, OH, United States; ^4^ Department of Systems Biology and Physiology, College of Medicine, University of Cincinnati, Cincinnati, OH, United States; ^5^ Department of Pediatrics, College of Medicine, University of Cincinnati, Cincinnati, OH, United States

**Keywords:** cecal ligation and puncture, colivelin, endothelial injury, glycocalyx, organ injury

## Abstract

Endothelial dysfunction plays a central role in the pathogenesis of sepsis-mediated multiple organ failure. Several clinical and experimental studies have suggested that the glycocalyx is an early target of endothelial injury during an infection. Colivelin, a synthetic derivative of the mitochondrial peptide humanin, has displayed cytoprotective effects in oxidative conditions. In the current study, we aimed to determine the potential therapeutic effects of colivelin in endothelial dysfunction and outcomes of sepsis *in vivo.* Male C57BL/6 mice were subjected to a clinically relevant model of polymicrobial sepsis by cecal ligation and puncture (CLP) and were treated with vehicle or colivelin (100-200 µg/kg) intraperitoneally at 1 h after CLP. We observed that vehicle-treated mice had early elevation of plasma levels of the adhesion molecules ICAM-1 and P-selectin, the angiogenetic factor endoglin and the glycocalyx syndecan-1 at 6 h after CLP when compared to control mice, while levels of angiopoietin-2, a mediator of microvascular disintegration, and the proprotein convertase subtilisin/kexin type 9, an enzyme implicated in clearance of endotoxins, raised at 18 h after CLP. The early elevation of these endothelial and glycocalyx damage biomarkers coincided with lung histological injury and neutrophil inflammation in lung, liver, and kidneys. At transmission electron microscopy analysis, thoracic aortas of septic mice showed increased glycocalyx breakdown and shedding, and damaged mitochondria in endothelial and smooth muscle cells. Treatment with colivelin ameliorated lung architecture, reduced organ neutrophil infiltration, and attenuated plasma levels of syndecan-1, tumor necrosis factor-α, macrophage inflammatory protein-1α and interleukin-10. These therapeutic effects of colivelin were associated with amelioration of glycocalyx density and mitochondrial structure in the aorta. At molecular analysis, colivelin treatment was associated with inhibition of the signal transducer and activator of transcription 3 and activation of the AMP-activated protein kinase in the aorta and lung. In long-term outcomes studies up to 7 days, co-treatment of colivelin with antimicrobial agents significantly reduced the disease severity score when compared to treatment with antibiotics alone. In conclusion, our data support that damage of the glycocalyx is an early pathogenetic event during sepsis and that colivelin may have therapeutic potential for the treatment of sepsis-associated endothelial dysfunction.

## Introduction

Sepsis is a life-threating organ dysfunction caused by dysregulated host responses to infection (1). A recent global study reported 49 million cases and 11 million sepsis-related deaths in 2017, accounting for approximately 20% of all annual deaths globally (2). Endothelial injury is a hallmark of systemic inflammatory response syndrome during sepsis and largely contributes to the serious clinical consequences of the infection such as increased vascular permeability, tissue edema, augmented adhesion of leukocytes and platelet aggregation, and loss of flow-dependent vasodilation leading to profound decrease in systemic vascular tone, and collapse of the microcirculation, and contributing to acute lung, kidney and liver injury (3, 4).

Clinical and experimental studies have proven that the glycocalyx is one of the earliest sites involved during the pathogenesis of endothelial injury (5). The glycocalyx is a gel-like mesh layer which covers the luminal surface of vascular endothelial cells. It is composed of membrane-attached proteoglycans, glycosaminoglycan sidechains, glycoproteins, and adherent plasma proteins such as albumin and antithrombin. This structure is known to play critical roles in maintaining hemostasis (6) and coagulation, regulating leukocyte adhesion and rolling (7, 8), and sensing mechanical forces, such as shear stress and pressure (9). It also shields cell surface receptors and can prevent their activation by presenting a physical barrier. In sepsis, there is a distinct alteration in the composition of the endothelial glycocalyx following the activation of proteases, such as metalloproteinases, heparanase, and hyaluronidase, by bacterial and inflammatory insults (10). These enzymes lead to glycocalyx degradation *via* release of glycosaminoglycan sidechains, and if severe enough, loss of core membrane proteins. As the glycocalyx is shed, circulating levels of glycocalyx components, including syndecans, can be measured and are considered biomarkers of endothelial injury (11).

Mitochondria have emerged as important players in maintaining vascular homeostasis (12). In addition to energy production, mitochondria affect a variety of complex processes including inflammation and cell survival (13). Mitochondria-derived peptides, including humanin, encoded by short open reading frame in the mitochondrial DNA (mtDNA), have been recently described to have biological effects (14, 15). Several experimental studies describe potent cytoprotective effects of humanin and its synthetic derivatives (15). For example, humanin is shown to protect endothelial cells from oxidative stress (16, 17) and to prevent glucose-induce endothelial expression of adhesion molecules and apoptosis (18, 19). At the molecular level, humanin appears to regulate metabolic homeostasis through involvement of the signal transducer and activator of transcription 3 (STAT3) (20–22) and AMP-activated protein kinase (AMPK) (15, 23, 24). Recently, colivelin, a new generation potent humanin derivative has also been reported to have cytoprotective effects by inhibiting apoptosis and inflammatory response *in vitro* and *in vivo* models of neuronal degeneration and ischemic injury (25–28). Despite the substantial literatures on colivelin-mediated beneficial effects in neurological diseases, the effect of colivelin on the endothelial damage during a systemic inflammation, like sepsis, has not been investigated.

In the present study, by employing a clinically relevant mouse model of sepsis we hypothesized that endothelial damage occurs early during sepsis and is characterized by structural damage of glycocalyx and associated with organ dysfunction. We also sought to evaluate the therapeutic efficacy of colivelin in sepsis and its potential molecular mechanisms of action.

## Materials and methods

### Murine model of polymicrobial sepsis

The investigation conformed to the National Institutes of Health Guide for the Care and Use of Laboratory Animals (Eighth Edition, 2011) and was approved by the Institutional Animal Care and Use Committee of the Cincinnati Children’s Hospital Medical Center. Male C57BL/6 mice were obtained from Charles River Laboratories International, Inc. (Wilmington, MA). Mice were used at 3-5 months of age to mimic the equivalent for human ranges from 20 - 25 years (29). Male mice only were chosen for the experimentation to avoid interference from female hormonal fluctuations in sepsis responses during the estrous cycle. Mice were housed in pathogen-free conditions under a 10-h light/14-h dark cycle with free access to food and water. Mice were anesthetized with 2.0% isoflurane in 50% oxygen and polymicrobial sepsis was induced by cecal ligation and puncture (CLP) (30). After a midline laparotomy, the cecum was exteriorized, ligated and punctured twice with a 23-G needle. The cecum was then returned into the peritoneal cavity and the abdominal incision was closed. After the procedure, mice were randomly assigned to three treatment groups: a vehicle-treated group received distilled water (200 µl/mouse) intraperitoneally (i.p.); a 100 µg colivelin-treated group received the colivelin at 100 µg/kg i.p., and a 200 µg colivelin-treated group received the colivelin at 200 µg/kg i.p. at 1 h after CLP. The intraperitoneal injection was chosen to allow for a rapid uptake and bioavailability of the peptide. All groups of mice also received fluid resuscitation (35 ml/kg normal saline solution subcutaneously) immediately after, at 3 h and 12 h after the CLP procedure. To minimize pain at the surgical incision site, lidocaine hydrochloride (1%, 4 mg/kg total dose) was applied locally immediately after the procedure. Control mice did not undergo any surgical procedure; sham mice underwent laparotomy only without CLP. Mice were then sacrificed at 0, 6 and 18 h after CLP. Blood, lungs, kidneys, liver, and thoracic aortas were collected for biochemical assays.

### Long-term studies of severity of sepsis

In a separate study, another cohort of mice was subjected to the CLP procedure and was used for assessing health and moribundity conditions, and survival rate up to 7 days. Mice were divided into two treatment groups in a blind and random fashion: a vehicle-treated group received distilled water (200 µl/mouse), and a colivelin-treated group received colivelin (100 µg/kg subcutaneously) at 1 h, 3 h and 24 h after the CLP procedure. Twelve animals were included for each group. One animal was sacrificed because of unintentional extensive damage in the small intestine during surgical procedure and was excluded from the study. The subcutaneous injection was chosen to avoid further stress in the peritoneum since the animals also received intraperitoneal treatment of antibiotics. To mimic the clinical management of antimicrobial coverage, all mice received ceftriaxone (25 mg/kg) and metronidazole (12.5 mg/kg) intraperitoneally every 12 h after CLP for three days. To minimize pain, buprenorphine (0.05 mg/kg) was administered subcutaneously at 1 h after surgery and every 12 h for three days after surgery. All groups of mice also received fluid resuscitation (35 ml/kg normal saline with 5% dextrose subcutaneously) every 24 h for all the duration of the experimental period. Although some spontaneous death occurred given the acute severity of the disease, spontaneous death was not considered as endpoint for this study for ethical reasons. Animals were euthanized when they exhibited signs of moribundity. During the monitoring period a score system was developed according to the clinical signs of peritoneal sepsis (31, 32). Physical examination focused on four parameters: posture, feces consistency, eye appearance, hair coat. For each parameter a score 0 to 3 was given according to the abnormalities. Specifically, a score of 0 represents no symptoms; score of 1 represents minimum symptoms (awkward gait, loose stools, some watery ocular discharge, fuzzy facial fur); score of 2 represents mild symptoms (hunched or slow walk, watery stools, some yellow ocular discharge, rough hair coat); score of 3 represents severe symptoms (complete inability to move or lethargy, hemorrhagic diarrhea, red eyes with thick ocular discharge, pilo-erection). Weight loss of more than 20% was also considered a humane endpoint. Monitoring and weighing of the animals was performed daily by the laboratory personnel blinded to the treatment protocol and logged in a score sheet. Animals with cumulative scores >8 or weight loss > 20% from the initial body weight were euthanized. Therefore, mice experiencing spontaneous death or euthanized within 7 days were defined as non-survivor mice. Animals that survived the entire observation period of 7 days were also euthanized and were defined as survivor mice.

### Myeloperoxidase activity

Myeloperoxidase (MPO) activity was measured as an indicator of neutrophil infiltration in lung, kidneys and liver tissue (33). Tissues were homogenized in a solution containing 0.5% hexa-decyl-trimethyl-ammonium bromide dissolved in 10 mM potassium phosphate buffer (pH 7.0) and centrifuged for 30 min at 4000 × g at 4°C. An aliquot of the supernatant was allowed to react with a solution of tetra-methyl-benzidine (1.6 mM) and hydrogen peroxide (0.1 mM). The rate of change in absorbance was measured by spectrophotometry at 650 nm. MPO activity was defined as the quantity of enzyme degrading 1 μmol of hydrogen peroxide/min at 37°C and expressed in units per 100 mg weight of tissue.

### Histopathologic analysis

Paraffin-embedded sections of thoracic aortas and lungs were stained with hematoxylin and eosin for morphological evaluation by three independent observers blinded to the treatment groups. Lung injury was also analyzed by a semiquantitative score based on the following histologic features: alveolar capillary congestion, infiltration of red blood cells and inflammatory cells into the airspace, alveolar wall thickness, and hyaline membrane formation (34). A score of 0 represented normal findings and scores of 1, 2, 3 and 4 represented minimal (<25% lung involvement), mild (25-50% lung involvement), significant (50-75% lung involvement) and severe (>75% lung involvement) injury, respectively. The four variables were summed to represent the lung injury score (total score, 0–16).

### Transmission electron microscopy

Glycocalyx structure was assessed by transmission electron microscopy (35). At 6 h after CLP, mice were again anesthetized with 2.0% isoflurane in 50% oxygen and perfused *via* cardiac puncture with a solution for lanthanum staining composed of 2% glutaraldehyde, 2% sucrose, 0.1 M sodium cacodylate buffer (pH 7.3), and 2% lanthanum nitrate. Thereafter, the aorta was harvested and diced in three to four pieces of approximately 1 mm^3^ each. Sections were immersed for 2 h in the lanthanum staining solution and then immersed overnight in a solution composed of 2% sucrose and 0.1 M sodium cacodylate buffer (pH 7.3). After washing in alkaline 2% sucrose and 0.03 M NaOH solution, sections were immersed in 2% osmium tetroxide and 2% lanthanum nitrate, embedded and cut with ultramicrotome. The sections were viewed and photographed on Hitachi H-7650 transmission electron microscope at 120 kV.

### Plasma levels of cytokines and biomarkers of endothelial injury

Plasma levels of tumor necrosis factor-α (TNFα), interleukin (IL)-1β, IL-6, IL-10, keratinocytes-derived chemokine (KC), and macrophage inflammatory proteins (MIP-1α) were used as indices of the systemic inflammatory response and were evaluated by a commercially available multiplex array system (Milliplex, Millipore Corporation, Billerica, MA). Plasma levels of endoglin, intercellular adhesion molecule-1 (ICAM-1), P-selectin, proprotein convertase subtilisin/kexin type 9 (PCSK9), and angiopoietin-2 (Ang 2) were used as indices of endothelial injury and were evaluated by a commercially available multiplex array system (R&D Systems, Minneapolis, MN). Plasma levels of syndecan-1 were used as indices of glycocalyx damage and were evaluated by a mouse syndecan-1 sandwich-type enzyme-linked immunosorbent assay (ELISA) kit (Boster Biological Technology Co., California, US). Assays were performed using the protocols recommended by the manufacturer.

### Subcellular fractionation

Subcellular fractionation was performed using a centrifugation model. Tissue samples of lung and thoracic aortas were homogenized in a buffer (50 mg tissue/100 µL) containing 0.32 M sucrose, 10 mM Tris-HCl (pH 7.4), 1 mM EGTA, 2 mM EDTA, 5 mM NaN3, 10 mM β-mercaptoethanol, 2 µM leupeptin, 0.15 µM pepstatin A, 0.2 mM phenylmethanesulfonyl fluoride, 50 mM NaF, 1 mM sodium orthovanadate and 0.4 nM microcystin. Samples were centrifuged at 1000x g for 10 min at 4°C and the supernatants collected as cytosol extracts, which also contain membrane and mitochondria. The pellets were then solubilized in Triton buffer (1% Triton X-100, 250 mM NaCl, 50 mM Tris HCl at pH 7.5, 3 mM EGTA, 3 mM EDTA, 1 mM phenylme-thanesulfonyl fluoride, 0.1 mM sodium orthovanadate, 10% glycerol, 2 mM p-nitrophenyl phosphate, 0.5% NP-40 and 46 µM aprotinin). The lysates were rocked for 1 h and subsequently centrifuged at 15,000x g for 30 min at 4° C and the supernatant collected as nuclear extracts. The Bradford protein assay was then used for quantitative determination of total proteins.

### Western blot analysis

Cytosol content of total AMPKα1/α2 and its phosphorylated form pAMPKα1/α2 (Santa Cruz Biotechnology, Dallas, TX, USA), cytosol and nuclear content of STAT3 and its phosphorylated forms pSTAT3(Tyr705) and pSTAT3(Ser727) (Cell Signaling Technology, Danvers, MA, USA) were determined by immunoblot analyses; β-actin was concomitantly probed with mouse anti-β-actin (Santa Cruz Biotechnology) as a loading control for both cytosol and nuclear proteins. Extracts were heated at 70°C in equal volumes of 4x Protein Sample Loading Buffer. Twenty-five μg of proteins were loaded per lane on a 10% Bis-Tris gel. Proteins were separated electrophoretically and transferred to nitro-cellulose membranes. The immunoreaction was detected by near-infrared fluorescence. Membranes were blocked with Odyssey blocking buffer (LI-COR Biotechnology, Lincoln, NE, USA) and incubated with primary antibodies. Membranes were washed in PBS with 0.1% TWEEN 20 and incubated with near infrared fluorescent dye-conjugated secondary antibodies (IRDye goat anti-rabbit and anti-mouse IgG; LI-COR Biotechnology). Immunoblotting was performed by using the IBind Flex Western System (Thermo Fischer Scientific, Waltham, MA, USA) that uses sequential lateral flow to perform blocking and antibody binding. The Odyssey LI-COR scanner (LI-COR Biotechnology) was used for detection. Fold changes of relative intensity of proteins were calculated *versus* mean value of control mice upon data normalization with β-actin by NIH ImageJ 1.53k software (36). Normalization and quantification for AMPKα1/α2 was also validated by Revert total protein stain and Empiria Studio analysis (LI-COR Biotechnology).

### Materials

Unless otherwise stated, all chemicals were obtained from Sigma-Aldrich (St. Louis, MO).

### Statistical analysis

Statistical analysis was performed using SigmaPlot 14.0 (Systat Software, San Jose, CA, USA). Data in figures and text are expressed means ± SEM or median with 25th and 75th percentile of n observations (n = 3–17 animals for each group). The results were examined by analysis of variance followed by the Student–Newman–Keuls correction *post hoc* t-test. Statistical analysis of damage scores was performed using the non-parametric Mann–Whitney test. The Gehan-Breslow test was used to compare differences in survival rates (n = 11-12 animals for each group). A value of P<0.05 was considered significant.

## Results

### Glycocalyx shedding and endothelial damage occur early during polymicrobial sepsis and are associated with lung injury

**Figure 1:**
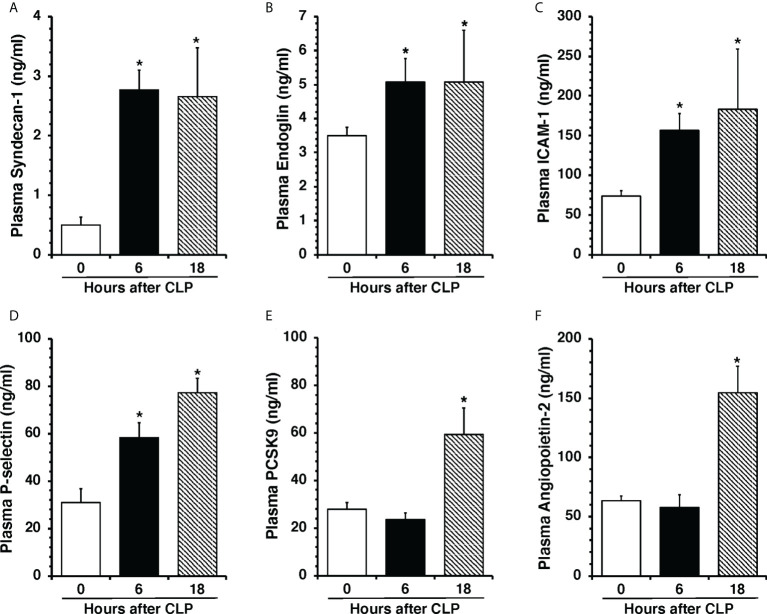
Plasma levels of Syndecan-1 **(A)**, Endoglin **(B)**, ICAM-1 **(C)**, P-selectin **(D)**, PCSK9 **(E)**, and Angiopoietin-2 **(F)** at 0 h, 6 h and 18 h after cecal ligation and puncture (CLP). Data represents the mean ± SEM of 10-13 mice for group (n=10 control group at 0 h, n=13 at 6 h, n=11 at 18 h). *Represents *P* < 0.05 *versus* control mice at time 0.

**Figure 2:**
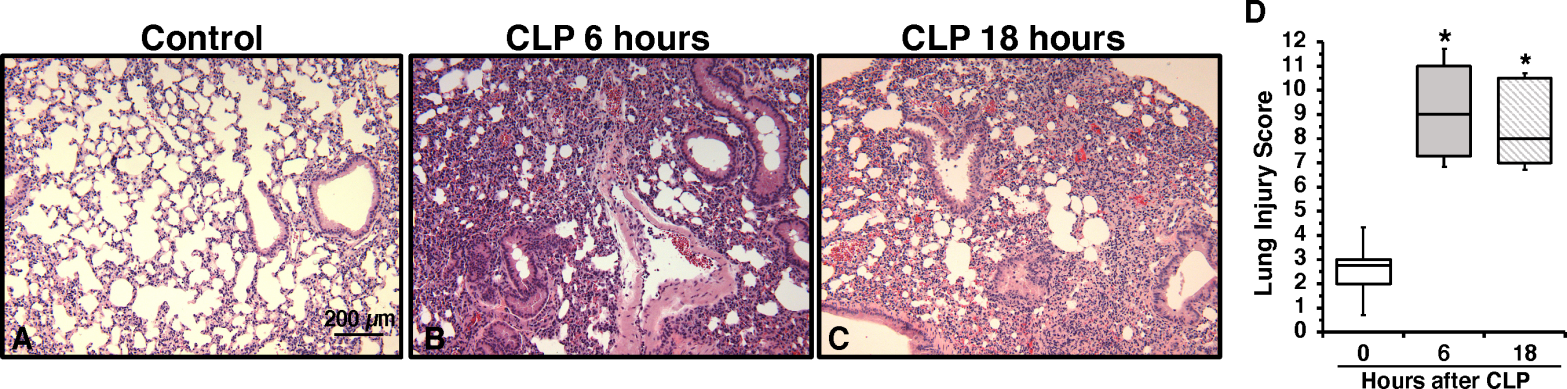
Representative histology photomicrographs of lung sections of a control mouse **(A)** or mice subjected to polymicrobial sepsis at 6 h **(B)** and 18 h **(C)** after cecal ligation and puncture (CLP). Lung damage at 6 h and 18 h after CLP was characterized by severe reduction of alveolar space, neutrophil adhesion along vascular wall, hemorrhage, and infiltration of inflammatory cells. Magnification x100. A similar pattern was seen in tissue sections of n=5 mice in each experimental group. **(D)** Histopathologic scores of lung sections (n=5 mice for each group). Lung injury was scored from 0 (no damage) to 16 (maximum damage). Box plots represent 25^th^ percentile, median, and 75^th^ percentile; error bars define 10^th^ and 90^th^ percentiles. *Represents *P* < 0.05 *versus* control mice at time 0.

To determine the onset of endothelial damage, we performed histology of thoracic aortas and we measured plasma biomarkers at 6 h and 18 h after CLP. Hematoxylin and eosin-stained sections of the thoracic aorta did not reveal alteration of cellular density or irregularities in the tunica intima, tunica media, and adventitia layers at 6 h or 18 h after CLP (**Supplementary Figure S1**). However, an early elevation of plasma levels of syndecan-1, a marker of glycocalyx breakdown and shedding, was observed at 6 h in mice subjected to CLP when compared to control mice at baseline conditions (2.77 ± 0.34 *versus* 0.49 ± 0.13 ng/ml, P<0.05; **Figure 1A**). This early glycocalyx damage was also associated with an early increase of the angiogenetic factor endoglin (5.07 ± 0.69 ng/ml), the adhesion molecules ICAM-1 (156.72 ± 20.93 ng/ml) and P-selectin (58.26 ± 6.40 ng/ml) when compared to control mice (3.50 ± 0.24, 73.96 ± 6.62, and 31.04 ± 5.73 ng/ml, respectively; P<0.05). At 18 h after CLP plasma syndecan-1, endoglin, ICAM-1 and P-selectin were still maintained at high levels (**Figures 1B–D**). At 18 h after CLP, septic mice also exhibited higher plasma levels of angiopoietin-2 (154.80 ± 21.90 ng/mL), a mediator of microvascular disintegration, and levels of PCSK9 (59.27 ± 11.28 ng/mL), an enzyme implicated in low-density lipoprotein receptor degradation and clearance of endotoxins, when compared to control mice (63.19 ± 4.08 and 27.99 ± 2.80, respectively; P<0.05) (**Figures 1E, F**). Early degradation of endothelial glycocalyx was also associated with higher lung injury score at 6 h, which persisted at 18 h after CLP, and was characterized by reduced alveolar space, and accumulation of red and inflammatory cells when compared to control mice at basal condition (**Figure 2**). To distinguish whether early endothelial damage was secondary to specific sepsis-induced immune response, we also measured these circulating biomarkers in sham mice, which underwent laparotomy but not CLP. In sham mice at 6 h, levels were not significantly different when compared with baseline levels of control mice. Sham mice at 18 h exhibited a significant elevation of P-selectin, PCSK9 and angiopoietin-2 (**Supplementary Figure S2**). There was only a mild infiltration of neutrophil, as determined by MPO activity, in the lung at 6 h when compared with control mice, but levels were significantly lower than mice subjected to CLP (**Supplementary Figure S3**). Thus, these data suggested that the early occurrence of endothelial damage is a specific sepsis-induced response and not induced by the sterile inflammation caused by the surgical procedures.

### Treatment with colivelin reduces neutrophil infiltration in lung, liver and kidney after CLP in a dose-independent manner

**Figure 3:**
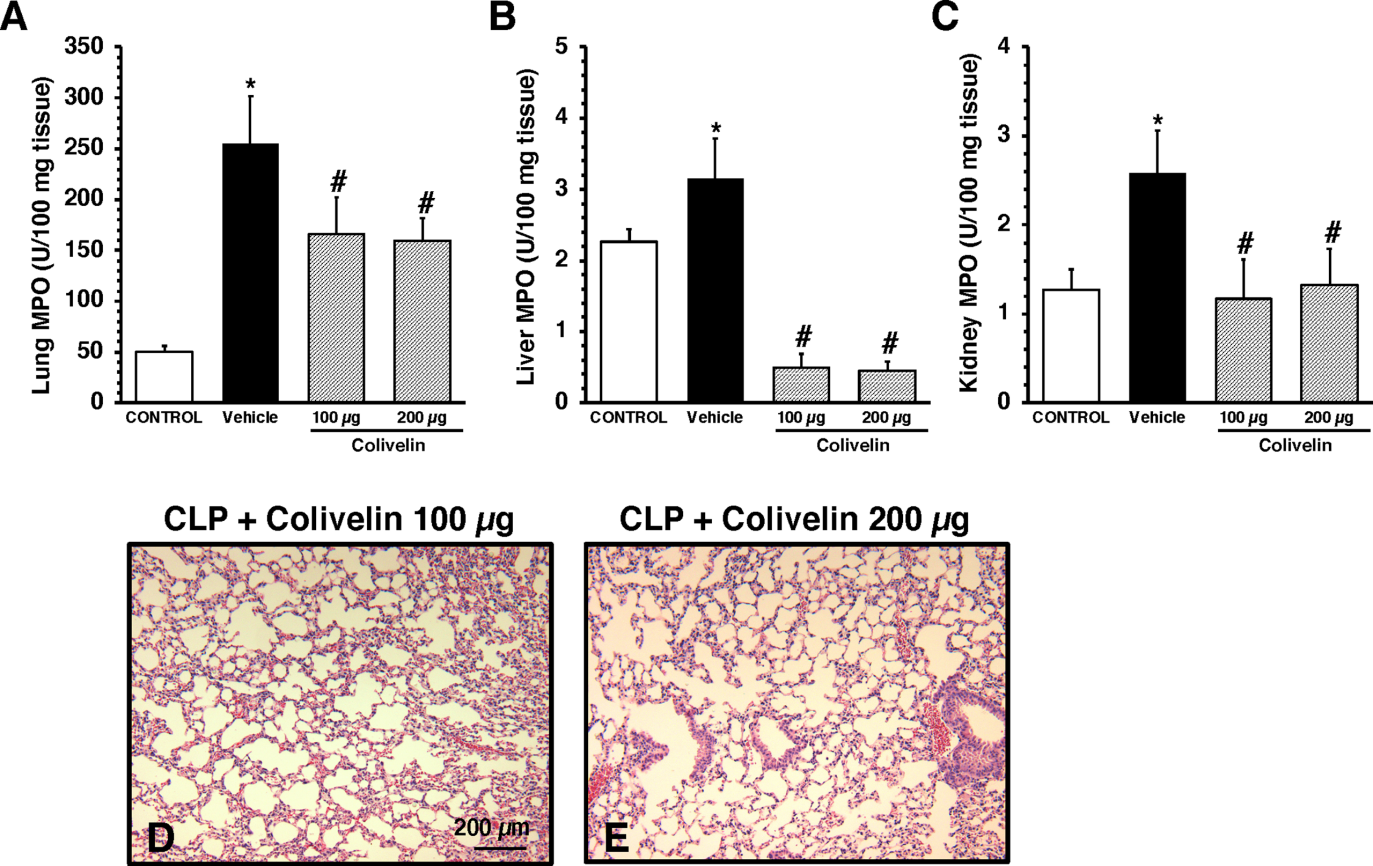
Activity of myeloperoxidase (MPO) in lung **(A)**, liver **(B)**, kidney **(C)** at 6 h after cecal ligation and puncture (CLP). Data represents the mean ± SEM of 7-17 mice for group (n=17 control group, n=13 vehicle group, n=7 colivelin 100 µg group, n=11 colivelin 200 µg group). *Represents *P* < 0.05 *versus* control mice; #represents *P* < 0.05 *versus* vehicle-treated mice. **(D-E)** Representative histology photomicrographs of lung sections of colivelin-treated mice at 6 h after CLP. Vehicle (200 µl distilled water) or colivelin (100 or 200 µg/kg) was administered intraperitoneally at 1 h after CLP. Magnification x100. A similar pattern was seen in tissue sections of n=5 mice in each experimental group.

Considering the early elevation in plasma levels of adhesion molecules, we next determined the effects of treatment with the peptide colivelin on neutrophil infiltration by measuring MPO activity in major organs at 6 h after CLP. Vehicle-treated mice had higher MPO activity in lungs, liver and kidneys when compared to control mice at basal conditions. Treatment with colivelin significantly decreased MPO activity in lungs, liver and kidneys in a dose-independent manner when compared to vehicle treatment (**Figures 3A–C**). Microscopic examination of hematoxylin and eosin-stained lung sections confirmed that treatment with colivelin reduced infiltration of inflammatory cells and ameliorated alveolar damage in the lung (**Figures 3D, E**) when compared to vehicle treatment (**Figure 2B**).

### Treatment with colivelin reduces plasma levels of cytokines after CLP in a dose-independent manner

**Figure 4:**
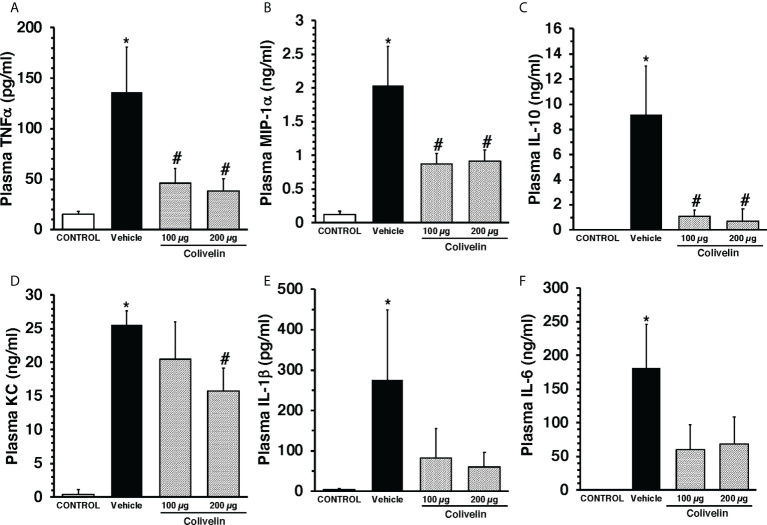
Plasma levels of TNFα **(A)**, MIP-1α **(B)**, IL-10 **(C)**, KC **(D)**, IL-1β **(E)**, and IL-6 **(F)** at 6 h after cecal ligation and puncture (CLP). Vehicle (200 µl distilled water) or colivelin (100 or 200 µg/kg) was administered intraperitoneally at 1 h after CLP. Data represents the mean ± SEM of 4-7 mice for group (n=4 control group, n=6 vehicle group, n=4 colivelin 100 µg group, n=7 colivelin 200 µg group). *Represents *P* < 0.05 *versus* control mice; #represents *P* < 0.05 *versus* vehicle-treated mice.

To evaluate the effect of colivelin on systemic inflammatory response, a panel of Th1/Th2/Th17 cytokines was measured. At 6 h after CLP, plasma levels of IL-1β, IL-6, IL-10, KC, TNF-α, and MIP-1α were significantly increased in vehicle-treated mice compared to control mice. Colivelin treatment significantly decreased levels of TNF-α, MIP-1α and IL-10 in a dose independent-manner. Levels of KC were significantly reduced in the mice treated with colivelin at 200 µg/kg. There was also a trend towards reduction of IL-1β and IL-6 after treatment with colivelin, but levels of these cytokines were not statistically different when compared with vehicle treatment (**Figure 4**).

### Treatment with colivelin ameliorates endothelial glycocalyx damage and mitochondrial damage in thoracic aortas after CLP

**Figure 5:**
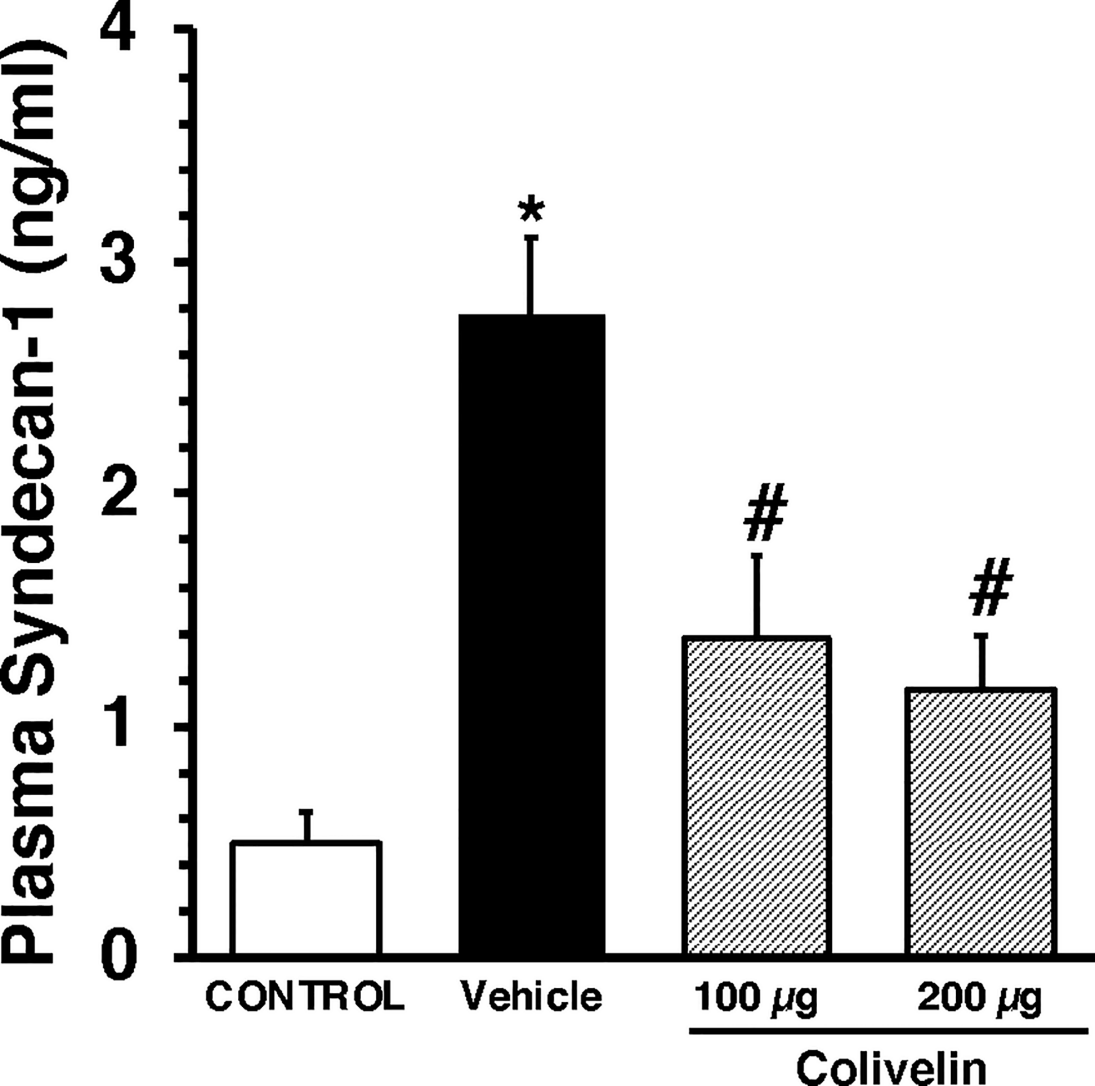
Plasma levels of Syndecan-1 at 6 h after cecal ligation and puncture (CLP). Vehicle (200 µl distilled water) or colivelin (100 or 200 µg/kg) was administered intraperitoneally at 1 h after CLP. Data represents the mean ± SEM of 7-13 mice for group (n=10 control group, n=13 vehicle group, n=7 colivelin 100 µg group, n=11 colivelin 200 µg group). *Represents *P* < 0.05 *versus* control mice; #represents *P* < 0.05 *versus* vehicle-treated mice.

**Figure 6:**
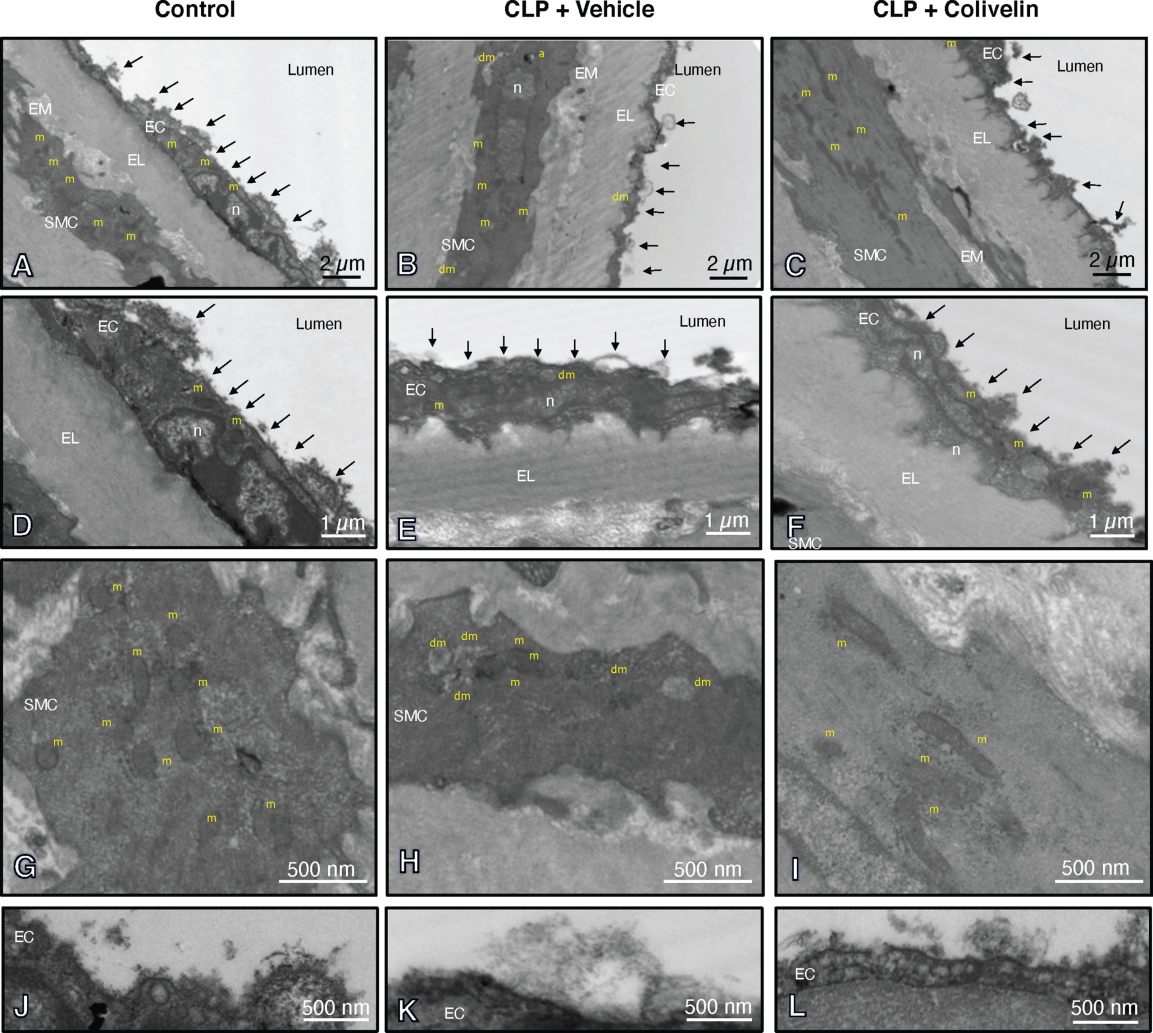
Transmission electron microscopy sections of thoracic aortas with lanthanum staining at 6 h after cecal ligation and puncture (CLP). Panels of control mice **(A, D, G, J)** show thick endothelial glycocalyx layer with dense individual bundles and normal mitochondria in endothelial and smooth muscle cells. Panels of vehicle-treated mice **(B, E, H, K)** show thin glycocalyx with bundles with loose structure and some damaged mitochondria and authophagic vesicles at 6 h after CLP. Panels of colivelin-treated mice **(C, F, I, L)** show well preserved thin glycocalyx and normal mitochondria at 6 h after CLP. Vehicle (200 µl distilled water) or colivelin (100 µg/kg) was administered intraperitoneally at 1 h after CLP. Arrows = glycocalyx; EC = endothelial cell; EL = elastica lamina; EM = extracellular matrix; RC = red cell; SMC = smooth muscle cell; a = authophagosome; n = nucleus; m = normal mitochondria; dm = damaged swollen mitochondria presenting translucent matrix and disrupted cristae.

We next evaluated the effect of colivelin on endothelial injury. Colivelin treatment significantly decreased levels of plasma syndecan-1 in a dose-independent manner at 6 h after CLP, thus suggesting reduction in glycocalyx shedding (**Figure 5**). Since effects of the peptide were in a dose-independent manner, we next examined the ultrastructural changes of the thoracic aortas in mice treated with colivelin at 100 µg/kg only (**Figure 6**). At electron microscopic analysis, mitochondria damage was evident in smooth muscle and endothelial cells in vehicle-treated mice at 6 h after CLP and was characterized by swollen mitochondria and presence of autophagosomes when compared to control mice. On the luminal surface the lanthanum staining showed a thick endothelial glycocalyx layer with dense individual bundles in control mice. At 6 h after CLP, the glycocalyx layer appeared thinner with less dense bundles with loose structure in vehicle-treated mice. On the contrary, in colivelin-treated mice mitochondria appeared normal with dense matrix in all cell types and the dense structure of glycocalyx appeared well preserved when compared to vehicle treatment (**Figure 6**).

### Treatment with colivelin inhibits STAT3 activation in thoracic aortas and lungs after CLP

**Figure 7:**
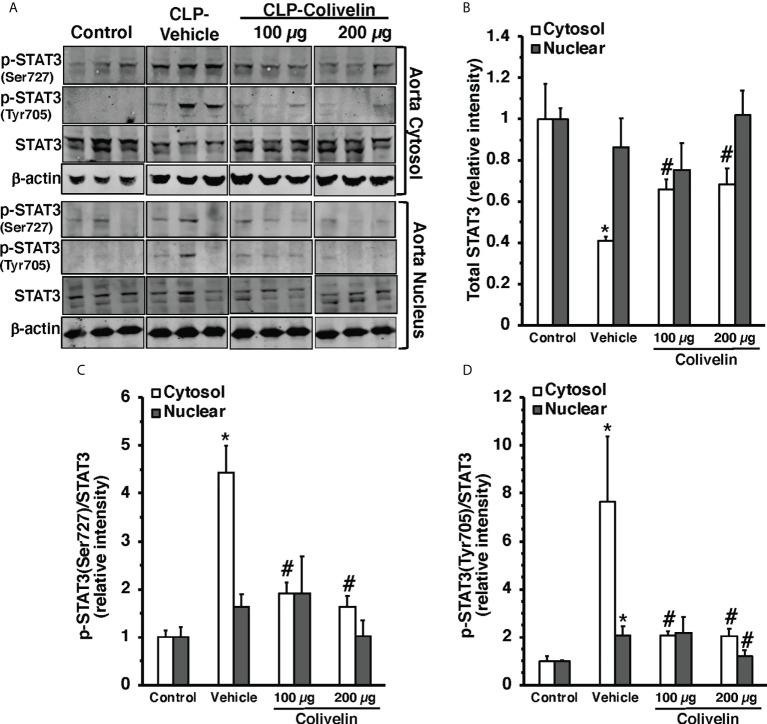
Representative Western blots of total STAT3, p-STAT3(Ser727) and p-STAT3(Tyr705) in cytosol and nuclear extracts of thoracic aorta; β-actin was used as loading control protein **(A)**. Image analyses of cytosol and nuclear of relative intensity of total STAT3 **(B)**, ratio of p-STAT3(Ser727)/STAT3 **(C)**, and ratio of p-STAT3(Tyr705)/STAT3 **(D)** as determined by densitometry. Vehicle (200 µl distilled water) or colivelin (100 µg/kg) was administered intraperitoneally at 1 h after CLP. Each data represents the mean ± SEM of 3-4 animals for each group. *Represents *P < 0.05* versus control mice; #represents *P < 0.05* versus vehicle-treated mice.

**Figure 8:**
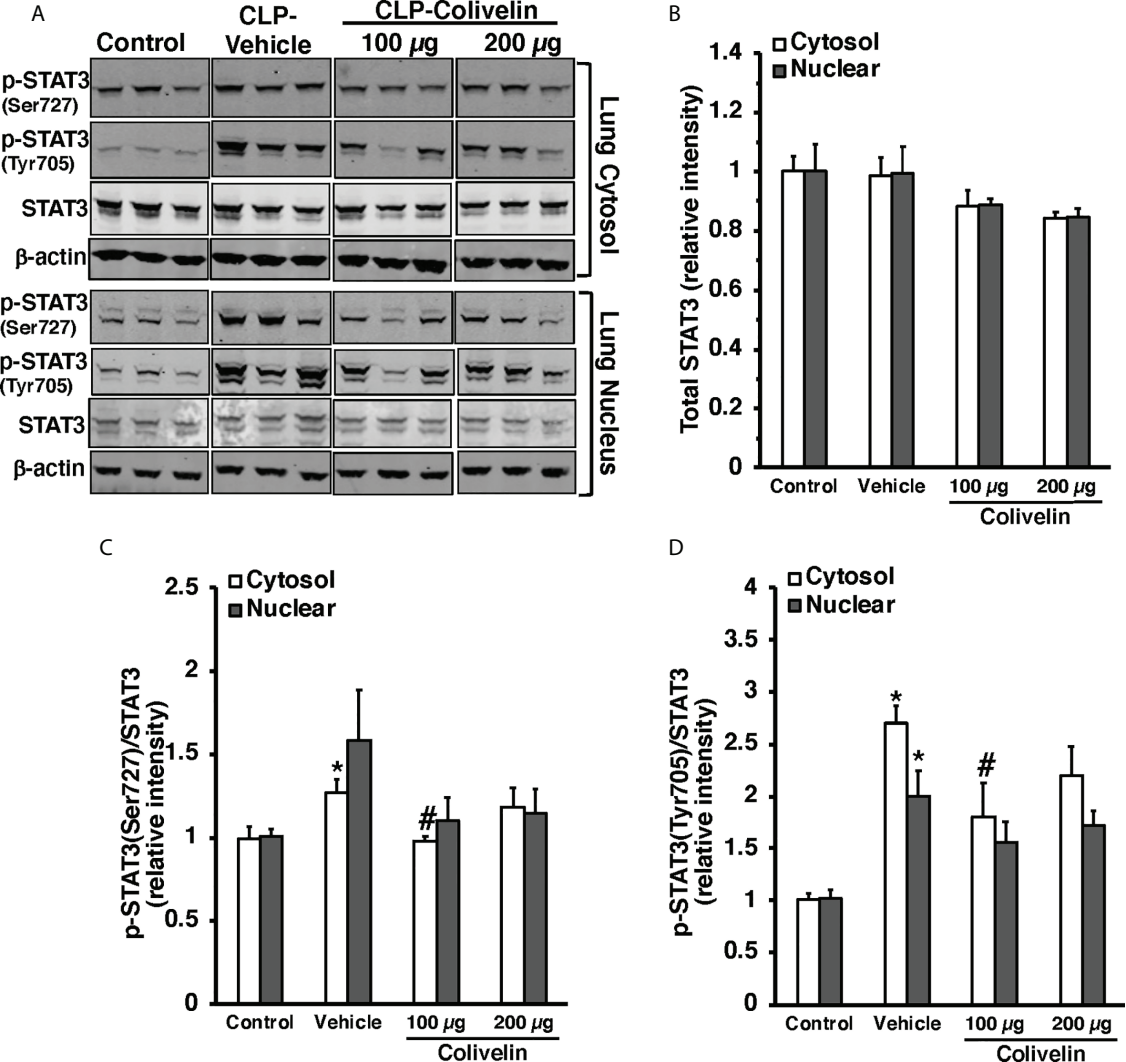
Representative Western blots of total STAT3, p-STAT3(Ser727) and p-STAT3(Tyr705) in lung cytosol and nuclear extracts; β-actin was used as loading control protein **(A)**. Image analyses of cytosol and nuclear of relative intensity of total STAT3 **(B)**, ratio of p-STAT3(Ser727)/STAT3 **(C)**, and ratio of p-STAT3(Tyr705)/STAT3 **(D)** as determined by densitometry. Vehicle (200 µl distilled water) or colivelin (100 µg/kg) was administered intraperitoneally at 1 h after CLP. Each data represents the mean ± SEM of 3-4 mice for group (n=3 control group, n=4 vehicle group, n=4 colivelin 100 µg group). *Represents *P < 0.05* versus control mice; #represents *P < 0.05* versus vehicle-treated mice.

Since colivelin has been reported to activate STAT3 *in vitro*, we next determined whether colivelin induced changes in STAT3 activation and intracellular localization in aortas and lungs. Control mice exhibited marginal levels of pSTAT3(Ser727), whereas the pSTAT3(Tyr705) was undetectable in both cytosol and nuclear compartments of thoracic aortas (**Figure 7**). At 6 h after CLP, the levels of total STAT3 were reduced in the cytosol while they remained unchanged in the nucleus in vehicle-treated mice when compared to control mice. On the contrary, the expression of pSTAT3(Ser727) was significantly upregulated in the cytosol, while there was a trend towards increase in the nucleus; pSTAT3(Tyr705) was significantly upregulated in the cytosol and nuclear compartments when compared to basal levels of control mice, thus suggesting an overall activation of the transcription factor after sepsis. Interestingly, in thoracic aortas of colivelin-treated mice, cytosolic expression of both pSTAT3(Ser727) and pSTAT3(Tyr705) was significantly reduced. Colivelin treatment did not affect nuclear expression of pSTAT3(Ser727), while it inhibited pSTAT3(Tyr705) at the highest dose. Furthermore, the levels of total STAT3 were restored in the cytosol while they remained unchanged in the nucleus in colivelin-treated mice when compared to vehicle treatment (**Figure 7**). In the lung, there was a constitutive expression of both pSTAT3(Ser727) and pSTAT3(Tyr705) in the cytosol and nuclear compartments of control mice (**Figure 8**). At 6 h after CLP, the expression of pSTAT3(Ser727) was significantly upregulated in the cytosol, while there was a trend towards increase in the nucleus (P=0.063); pSTAT3(Tyr705) was significantly upregulated in the cytosol and nuclear compartments when compared to basal levels of control mice, thus suggesting an overall activation of the transcription factor also in the lung after sepsis. Interestingly, in the lung of colivelin-treated mice, cytosolic expression of both pSTAT3(Ser727) and pSTAT3(Tyr705) was significantly reduced when compared to vehicle-treatment. Nuclear expression of both pSTAT3(Ser727) and pSTAT3(Tyr705) was lower than vehicle-treated mice, but not statistically significant. In the lung, levels of total STAT3 were similar among the three groups of mice (**Figure 8**).

### Treatment with colivelin activates AMPK in thoracic aortas after CLP

**Figure 9:**
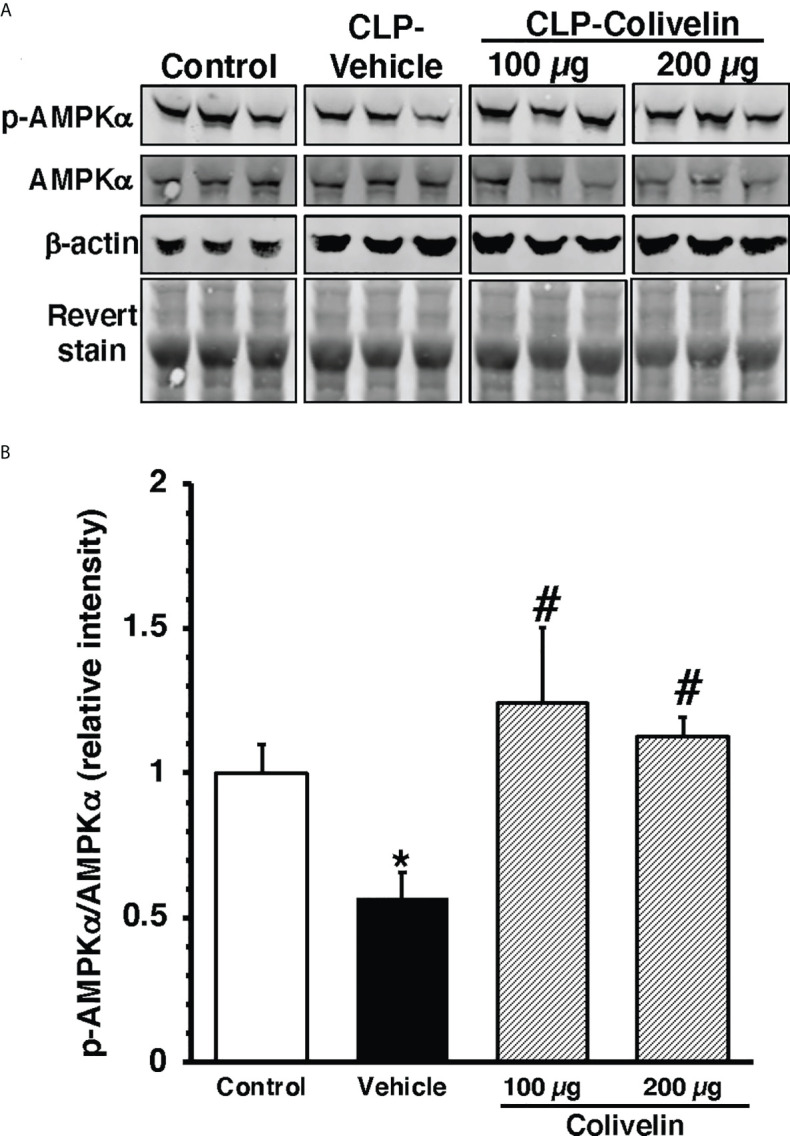
Representative Western blots of total AMPKα and pAMPKα in cytosol extracts of thoracic aorta; β-actin and Revert stain were used to verify loading of proteins **(A)**. Image analyses of ratio of relative intensity of p-AMPKα/AMPKα **(B)** as determined by densitometry. Vehicle (200 µl distilled water) or colivelin (100 µg/kg) was administered intraperitoneally at 1 h after CLP. Each data represents the mean ± SEM of 3-4 mice for group (n=3 control group, n=4 vehicle group, n=4 colivelin 100 µg group). *Represents *P < 0.05* versus control mice; #represents *P < 0.05* versus vehicle-treated mice.

To further examine the molecular mechanism of colivelin, we also determined the cytosolic activation of AMPK, the crucial regulator of mitochondrial control quality. At 6 h after CLP, the phosphorylated active pAMPKα1/α2 were reduced in the cytosol of thoracic aortas in vehicle-treated mice when compared to basal levels of control mice. Colivelin treatment significantly increased the ratio of the phosphorylated/total forms in a dose-independent manner, thus suggesting the restoration of the kinase function (**Figure 9**).

### Treatment with colivelin ameliorated long-term outcomes after CLP

**Figure 10:**
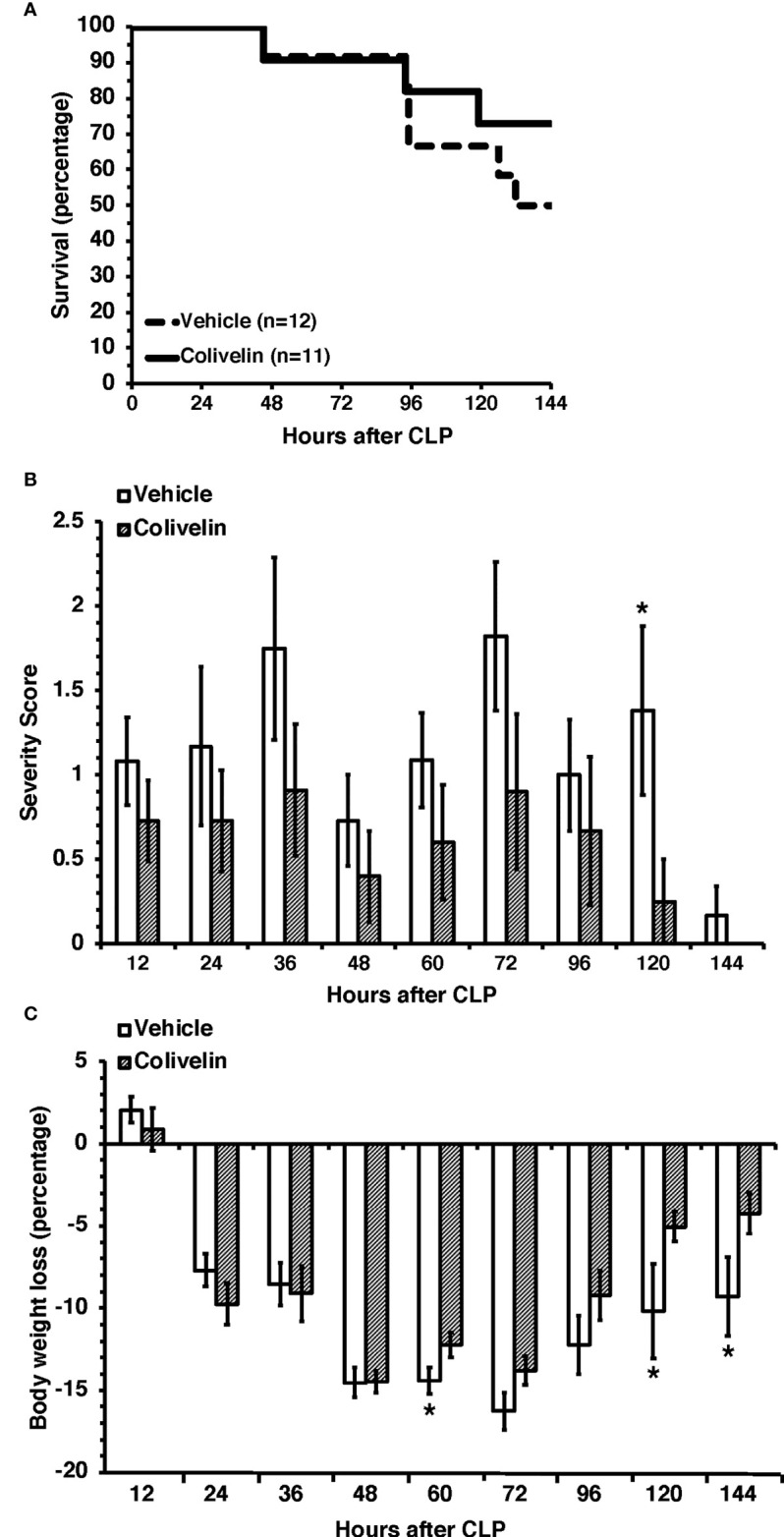
Survival rate **(A)**, severity score **(B)** and body weight loss **(C)** of mice at 7 days after cecal ligation and puncture (CLP). Mice were subjected to CLP and received colivelin (100 µg/kg subcutaneously) or vehicle at 1 h, 3 h and 24 h after CLP. All mice received fluid resuscitation (35 ml/kg normal saline with 5% dextrose subcutaneously) every 24 h up to 7 days and ceftriaxone (25 mg/kg) and metronidazole (12.5 mg/kg) intraperitoneally every 12 h up to 3 days after the CLP procedure. *Represents P < 0.05 versus colivelin-treated mice.

Given the early beneficial effects of colivelin on organ and endothelial injury induced by sepsis, we sought to determine the effect of the peptide in long-term outcomes. In long-term studies, septic mice were treated with colivelin (100 µg/kg subcutaneously) or vehicle at 1 h, 3 h and 24 h after CLP and were monitored up to 7 days. To mimic the clinical condition, all mice also received antibiotic therapy for three days and fluid resuscitation for all the duration of the experimental period. The vehicle-treated group exhibited a survival rate of 50% as 6 out of 12 mice survived at 7 days after CLP. The colivelin-treated group experienced a slight, but not significant, increase of survival rate (72.6%) as 8 out of 11 mice survived at 7 days (**Figure 10A**). Both vehicle- and colivelin-treated mice exhibited diarrhea, pilo-erection and signs of lethargy in the early 36 h after CLP. Symptoms declined at 48 h but increased again at later time after antibiotics discontinuation in both vehicle and colivelin-treated groups. However, colivelin-treated mice exhibited less severe signs of sepsis for all the duration of the observation period and survivor colivelin-treated mice were significantly healthier than survivor vehicle-treated mice at 6 and 7 days after CLP (**Figure 10B**). Both vehicle- and colivelin-treated mice experienced a similar body weight loss in the first two days after CLP. However, at later time points vehicle-treated mice maintained a significant lower weight than colivelin-treated mice (**Figure 10C**).

## Discussion

In the present work, we demonstrated that increased plasma levels of biomarkers of endothelial permeability, inflammation and adhesiveness occurred at the early stage of experimental sepsis in mice (i.e., at 6 h after CLP) and coincided with structural changes of endothelial glycocalyx in the aorta and with inflammation of major organs. We also demonstrated for the first time that colivelin, a potent synthetic humanin derivative, is a potential therapeutic compound to restore endothelial stability and improve outcomes of sepsis. We found, in fact, that colivelin treatment attenuated infiltration of inflammatory cells in lung, kidney and liver, reduced the systemic release of the pro-inflammatory cytokines, and, when given as an adjunctive treatment to the standard fluid resuscitation and antibiotics, improved long-term recovery and health conditions of septic mice.

Being responsible of important physiological functions, such as hemostasis, vasomotor control, barrier integrity and immunological function, the endothelium is a critical cellular system for host survival following severe injury, including sepsis (3, 4). Considering the systemic nature of sepsis, exposure to pathogen-associated molecular patterns and endogenous damage-associated molecular patterns may impair the structure and function of the endothelium and its glycocalyx layer. Several clinical studies have demonstrated elevated circulating levels of syndecan-1 as a marker of glycocalyx degradation in sepsis and are associated with organ dysfunction and mortality (37–40). Clinical studies have also profiled protein markers of endothelial activation in both the adult and pediatric populations and have reported significant associations with the severity of sepsis and septic shock, organ failure and mortality risks (41–44). Adhesion molecules, such as ICAM-1 and P-selectin, have been associated with poor outcomes of acute lung injury (45–47). In adult patients circulating P-selectin, measured at ICU admission, appear to be associated with sepsis development in time (48) and it may have diagnostic value for sepsis when used with other endothelial markers (40). Angiopoietin-2, which is produced in endothelial cells and pre-stored in the Weibel-Palade bodies, has been correlated with sepsis severity and death (42–44, 49) and with acute kidney injury and respiratory failure (50). Other novel biomarkers have been proposed for the evaluation of endothelial dysfunction (51). Endoglin, or CD105, is a membrane-bound glycoprotein that serves as a co-receptor for members of the transforming growth factor-β and functions as an angiogenetic factor. Although not yet determined in sepsis, circulating levels of soluble endoglin have been shown to be higher in the serum of patients with cardiovascular diseases with a significant inflammatory component (52, 53). Recent experimental and clinical studies have also supported a central role of PCSK9 in the clearance of pathogenic lipids such as the bacterial lipopolysaccharide (LPS) and in sepsis (54). Although mainly located in the liver PCSK9 is also expressed in vascular smooth muscle and endothelial cells and its expression is increased by stimulation with LPS (55), suggesting a critical role of PCSK9 in vascular function. Plasma PCSK9 levels have been shown to serve as a late biomarker of the severity of illness in patients with severe trauma injury in ICU (56) and sepsis (57) and have been correlated to endothelial dysfunction in patients with chronic kidney disease (58).

Despite these data on association with poor prognosis, the pathophysiology of glycocalyx injury and endothelial dysfunction and its potential role as therapeutic targets in improving sepsis outcomes remain unclear. In our study, we observed a distinct temporal profile of these circulating endothelial biomarkers and glycocalyx degradation. We observed that plasma elevation of the glycocalyx component syndecan-1 occurred early after CLP procedure and correlated with an early elevation of plasma levels of the adhesion molecules ICAM-1 and P-selectin. This early increased expression of circulating syndecan-1 support the hypothesis that the shedding of the glycocalyx concomitantly occurs with the critical period of the inflammatory process of the endothelium and may precede angiogenetic events as angiopoietin-2 was elevated only at 18 h after CLP. Interestingly, in our model we also have identified other novel markers, such as endoglin and PCSK9, whose pathophysiological role in sepsis deserves further investigation.

Neutrophil infiltration is a crucial pathophysiological event of organ injury. In normal conditions, adhesion molecules responsible for leukocyte adhesion are embedded in the glycocalyx and are shielded from leukocytes rolling. Therefore, shedding of the glycocalyx allows for neutrophil infiltration. In our model, the early increase of syndecan-1 and adhesion molecules temporally correlated with neutrophil infiltration in lung, liver and kidney. In this regard, it is noteworthy that elevation of circulating syndecan-1 was associated with inflammatory biomarkers of neutrophil activation, including MPO, and was predictive of adverse clinical outcomes in patients with sepsis due to pneumonia (59).

To restore the endothelial permeability barrier and improve outcome in sepsis, we tested the efficacy of colivelin, a new generation humanin peptide derivative (25). Humanin is a polypeptide containing 24 amino acids, which is encoded encoded by short open reading frame in the mtDNA and acts as retrograde signaling molecule to regulate inflammation (14). Humanin was first identified in the cDNA associated with neuroprotective effects in Alzheimer’s disease patients, and therefore recognized for its antiapoptotic properties (21). Previous *in vitro* studies have demonstrated that humanin has cytoprotective effects in human aortic endothelial cells against oxidative stress (16). A synthetic analogue with enhanced potency, humanin-G, has also been reported to inhibit cell death in high-glucose-induced apoptosis in human umbilical vein endothelial cells (17). Another potent humanin derivative is colivelin, a hybrid peptide named composed of activity-dependent neurotrophic factor and fused at the C-terminus to a fragment of humanin (25), which has been shown to provide beneficial effects in ischemia models *in vivo* (28). In our study, we demonstrated that *in vivo* treatment with colivelin reduced lung injury and reduced leukosequestration in lung, liver and kidney. These beneficial effects correlated with a significant reduction of circulating levels of syndecan-1, thus suggesting inhibition of glycocalyx shedding, when compared to vehicle treatment. To determine the beneficial effect of colivelin on glycocalyx and endothelium we comprehensively assessed the vascular damage of thoracic aortas by transmission electron microscopy and found that colivelin treatment was associated with amelioration of glycocalyx structure, as evidenced by the presence of thick and complex bundles when compared to the loose and thin structure in mice receiving vehicle. Although our analysis of the vascular wall was focused on glycocalyx structure, we also found that vascular damage in vehicle-treated mice was characterized by the presence of damaged mitochondria in both endothelial and smooth muscle cells. It must be considered that mitochondria in vascular smooth muscle and endothelial cells play a pivotal role in maintaining the structural integrity of the vascular wall, whereas their dysfunction leads to energy failure and contributes to inflammation *via* production of reactive oxygen species (12). In our study, colivelin treatment also ameliorated mitochondrial structure. Thus, taken together, our data demonstrated that the peptide affords multifactorial beneficial effects against oxidative and metabolic stress and against neutrophil adhesiveness and activation at the vascular level. Many preclinical and clinical studies have demonstrated an association between inflammatory cytokines and glycocalyx degradation biomarkers (35, 39, 60, 61). In our study colivelin treatment significantly blunted the systemic elevation of TNFα, MIP-1α, KC and IL-10, suggesting that the peptide interferes with the vicious cycle between impaired endothelial glycocalyx and neutrophil activation.

One of the most notable observations in our study was that treatment with colivelin improved long-term wellbeing outcomes of mice subjected to sepsis. To mimic human sepsis management, mice were resuscitated with fluids and treated with antibiotics. We observed that mice treated with a combination of fluids, antibiotics and colivelin experienced less severe clinical signs of sepsis up to 7 days after CLP when compared with animals treated only with fluids and antibiotics, suggesting beneficial effects of colivelin on recovery. The group of mice that received the adjunct therapy of colivelin also exhibited a higher, but not significant, survival rate (72.6%) than the group that received only vehicle in combinations with fluids and antibiotics (50%). However, it must be noted that for ethical reasons mortality was not used as endpoint of our study and we did not use a large number of mice; furthermore, some mice were euthanized according to criteria that predicted moribundity, thus, most probably affecting statistical significance.

In evaluating the molecular mechanisms of colivelin, we investigated the contribution of both STAT-3 and AMPK since these signaling pathways have been reported to be activated by humanin and its derivatives. STAT3 is a crucial transcription factor, which plays a role in development, inflammation, immunity, metabolism and cancer (62). In addition to its established role as a nuclear transcription factor, a pool of STAT3 has been described in the mitochondria. STAT3 in the mitochondria requires Ser727 but not Tyr705 phosphorylation and functions as a positive regulator of mitochondrial electron transport chain for ATP production (63). *In vitro* studies have shown that treatment with humanin and its analogues may exert protective functions through STAT3 phosphorylation (20, 23, 64). In a murine model of ischemic stroke, the beneficial effects on neuronal death and axonal remodeling of colivelin have also been associated with activation of STAT3 signaling (28). Previous studies have reported that expression of pSTAT3(Tyr705) increases in the lung, liver, and kidney in murine models of sepsis (65–67). However, these studies have not examined the subcellular localization of the different phosphorylated forms of STAT3. In our study, we observed for the first time that in addition to the lung, pSTAT3(Tyr705) is also activated in the cytosol and nuclear compartments of the aorta at 6 h after CLP in vehicle-treated mice. Interestingly, we also found that distinct subcellular localization of the pSTAT3(Ser727), which increased in both aortas and lungs and was preferentially located in the cytosol. It is important to note that non-canonical STAT3 activation through Ser727 phosphorylation has been recently demonstrated to serve as a crucial signaling intermediary for TLR4-induced glycolysis, macrophage metabolic reprogramming and inflammation (68). An intriguing finding of our study was that colivelin treatment inhibited the activation of STAT3 in the aorta and lung and was associated with improvement of endothelial damage and pulmonary protection. These data are in discrepancy with previous *in vivo* and *in vitro* studies demonstrating that colivelin may act as a potent activator of STAT3 (25, 28). A potential reason for this discrepancy on STAT3 activation by colivelin may be due to the different disease models. Our study is the first to investigate the beneficial effects in an infection condition, while previous *in vivo* and *in vitro* studies have focused on conditions of neurodegeneration in Alzheimer’s disease and ischemia and reperfusion injury models. On the contrary, our findings are consistent with previous studies demonstrating that inhibitors of STAT3, such as Stattic, ameliorate inflammatory responses in endotoxin-induced acute lung injury (69).

To further understand the molecular mechanisms of colivelin, we also investigated the contribution of AMPK signaling pathway. AMPK is a serine/threonine protein kinase, which is the crucial regulator of energy metabolism and mitochondrial quality control (70). In our study, we observed that the thoracic aorta of colivelin-treated mice had increased activation of AMPK. It must be noted that humanin and humanin analogues may exert beneficial effects in oxidative stress by activation of AMPK (71, 72). Furthermore, it has been proposed that AMPK activation exerts anti-inflammatory effects in endotoxic shock in mice by inhibiting STAT3 signaling (73). Thus, it is plausible that the molecular mechanisms of the protective effect of colivelin in sepsis may be related to increased AMPK, which in turn inhibits phosphorylation of STAT3. Our current findings also support our previous studies demonstrating that pharmacological activation of AMPK ameliorates organ injury in mice subjected to experimental sepsis (74, 75).

In our study, however, we did not investigate the direct mechanisms by which colivelin interferes with STAT3 or AMPK activation. Mitochondrial peptides have been described to interact with cell surface receptors, such as formylpeptide-like-1 receptor and insulin-like growth factor binding protein-3 (14). Specific *in vitro* studies in endothelial cells are, therefore, necessary to further establish the upstream molecular mechanisms of colivelin in preserving glycocalyx structure and function.

## Limitations

As a limitation of our study, we did not include a colivelin-treated control group of healthy mice. Furthermore, we did not investigate potential sex-differences in colivelin beneficial effects as we used only male mice. However, at this preliminary stage of our investigation, the main goal of our study was to evaluate the effect of colivelin post-treatment in a disease state without the phenotypic variability of the estrous cycle. Previous studies have investigated the effect of colivelin on healthy animals/cells in the context of experimental model of neurodegenerative diseases. In these studies, colivelin did not alter behavior parameters or cell viability (25). Further comprehensive studies and elucidations of hormonal influences are required to better understand the molecular mechanisms of this synthetic mitochondrial peptide.

Another limitation of our study is that we used two different routes of administration to explore the therapeutic efficacy of colivelin in short-term and long-term experiments. In the short-term studies, colivelin was injected intraperitoneally to allow for a rapid uptake and bioavailability of the peptide. However, for the wellness of the animals, we switched to subcutaneous injection for the long-term administration to avoid further stress in the peritoneum since the animals also required repetitive intraperitoneal injections of antibiotics. Thus, it can be speculated that differences in biodistribution might have resulted in less efficacy in long-term outcomes. However, it must be noted that the cytoprotective effects of colivelin or other humanin derivatives have been described through different routes of administration *in vivo* (76).

## Conclusion

In conclusion, our data indicates that endothelial dysfunction and glycocalyx damage are early events of lung injury in a murine model of polymicrobial sepsis. Treatment with the novel synthetic mitochondrial peptide colivelin exerted pulmonary protective effects and improved long-term recovery *via* activation of AMPK and inhibition of STAT3 in thoracic aortas and lung. With the ability to rescue endothelium function and ameliorate glycocalyx structure, colivelin should be investigated as adjunct therapy for the treatment of sepsis.

## Data availability statement

The raw data supporting the conclusions of this article will be made available by the authors, without undue reservation.

## Ethics statement

This study was reviewed and approved by Institutional Animal Care and Use Committee of the Cincinnati Children’s Hospital Medical Center.

## Author contributions

BZ conceived and designed the projects. CU contributed to the design of the study. CU and VW performed the animal experiments. CU, VW, GP, CP, MO and PL performed the biochemical assays. CU, GP, and BZ performed the electron microscopy analysis. CU, GP, and BZ analyzed the data and prepared graphics. CU and BZ wrote the draft of the manuscript. All authors contributed to the article and approved the submitted version.

## Funding

This work was supported by the National Institutes of Health grants R01 GM-067202 and GM-115973 to Basilia Zingarelli, and in part by grant P30 DK-078392 of the Digestive Research Core Center (Integrative Morphology Core). The content is solely the responsibility of the authors and does not necessarily represent the official views of the National Institutes of Health.

## Conflict of interest

The authors declare that the research was conducted in the absence of any commercial or financial relationships that could be construed as a potential conflict of interest.

## Publisher’s note

All claims expressed in this article are solely those of the authors and do not necessarily represent those of their affiliated organizations, or those of the publisher, the editors and the reviewers. Any product that may be evaluated in this article, or claim that may be made by its manufacturer, is not guaranteed or endorsed by the publisher.

## References

[1] Singer M Deutschman CS Seymour CW Shankar-Hari M Annane D Bauer M. The third international consensus definitions for sepsis and septic shock (Sepsis-3). JAMA (2016) 315:801–10. doi: 10.1001/jama.2016.0287 PMC4968574 26903338

[2] Rudd KE Johnson SC Agesa KM Shackelford KA Tsoi D Kievlan DR. Global, regional, and national sepsis incidence and mortality, 1990-2017: analysis for the global burden of disease study. Lancet (2020) 395:200–11. doi: 10.1016/S0140-6736(19)32989-7 PMC6970225 31954465

[3] Ince C Mayeux PR Nguyen T Gomez H Kellum JA Ospina-Tascón GA. ADQI XIV workgroup. the endothelium in sepsis. Shock (2016) 45:259–70. doi: 10.1097/SHK.0000000000000473 PMC5281063 26871664

[4] Joffre J Hellman J. Oxidative stress and endothelial dysfunction in sepsis and acute inflammation. Antioxid Redox Signal (2021) 35:1291–307. doi: 10.1089/ars.2021.0027 33637016

[5] Chelazzi C Villa G Mancinelli P De Gaudio AR Adembri C. Glycocalyx and sepsis-induced alterations in vascular permeability. Crit Care (2015) 19:26. doi: 10.1186/s13054-015-0741-z 25887223 PMC4308932

[6] Curry FE Adamson RH. Endothelial glycocalyx: permeability barrier and mechanosensor. Ann BioMed Eng (2012) 40:828–39. doi: 10.1007/s10439-011-0429-8 PMC5042904 22009311

[7] Lipowsky HH. The endothelial glycocalyx as a barrier to leukocyte adhesion and its mediation by extracellular proteases. Ann BioMed Eng (2012) 40:840–8. doi: 10.1007/s10439-011-0427-x PMC3306510 21984514

[8] Mulivor AW Lipowsky HH. Role of glycocalyx in leukocyte-endothelial cell adhesion. Am J Physiol Heart Circ Physiol (2002) 283:H1282–91. doi: 10.1152/ajpheart.00117.2002 12234777

[9] Tarbell JM Ebong EE. The endothelial glycocalyx: a mechano-sensor and -transducer. Sci Signal (2008) 1:pt8. doi: 10.1126/scisignal.140pt8 18840877

[10] Becker BF Jacob M Leipert S Salmon AH Chappell D. Degradation of the endothelial glycocalyx in clinical settings: searching for the sheddases. Br J Clin Pharmacol (2015) 80:389–402. doi: 10.1111/bcp.12629 25778676 PMC4574825

[11] Iba T Levy JH. Derangement of the endothelial glycocalyx in sepsis. J Thromb Haemost (2019) 17:283–94. doi: 10.1111/jth.14371 30582882

[12] Kirkman DL Robinson AT Rossman MJ Seals DR Edwards DG. Mitochondrial contributions to vascular endothelial dysfunction, arterial stiffness, and cardiovascular diseases. Am J Physiol Heart Circ Physiol (2021) 320:H2080–100. doi: 10.1152/ajpheart.00917.2020 PMC8163660 33834868

[13] Lionaki E Gkikas I Tavernarakis N. Differential protein distribution between the nucleus and mitochondria: Implications in aging. Front Genet (2016) 7:162. doi: 10.3389/fgene.2016.00162 27695477 PMC5025450

[14] Lee C Yen K Cohen P. Humanin: a harbinger of mitochondrial derived peptides? trends endocrinol. Metab (2013) 24:222–8. doi: 10.1016/j.tem.2013.01.005 PMC3641182 23402768

[15] Lee C Zeng J Drew BG Sallam T Martin-Montalvo A Wan J. The mitochondrial-derived peptide MOTS-c promotes metabolic homeostasis and reduces obesity and insulin resistance. Cell Metab (2015) 21:443–54. doi: 10.1016/j.cmet.2015.02.009 PMC4350682 25738459

[16] Bachar AR Scheffer L Schroeder AS Nakamura HK Cobb LJ Oh YK. Humanin is expressed in human vascular walls and has a cytoprotective effect against oxidized LDL-induced oxidative stress. Cardiovasc Res (2010) 88:360–6. doi: 10.1093/cvr/cvq191 PMC2952532 20562421

[17] Shi D Zhou X Wang H. S14G-humanin (HNG) protects retinal endothelial cells from UV-b-induced NLRP3 inflammation activation through inhibiting egr-1. Inflammation Res (2021) 70:1141–50. doi: 10.1007/s00011-021-01489-4 34459932

[18] Wang X Wu Z He Y Zhang H Tian L Zheng C. Humanin prevents high glucose-induced monocyte adhesion to endothelial cells by targeting KLF2. Mol Immunol (2018) 101:245–50. doi: 10.1016/j.molimm.2018.07.008 30029058

[19] Xie Y Liu ZH Li XY Zhou YD Xu X Hu LF. Protection effect of [Gly14]-humanin from apoptosis induced by high glucose in human umbilical vein endothelial cells. Diabetes Res Clin Pract (2014) 106:560–6. doi: 10.1016/j.diabres.2014.09.020 25451915

[20] Hoang PT Park P Cobb LJ Paharkova-Vatchkova V Hakimi M Cohen P. The neurosurvival factor humanin inhibits β-cell apoptosis *via* signal transducer and activator of transcription 3 activation and delays and ameliorates diabetes in non-obese diabetic mice. Metabolism (2010) 59:343–9. doi: 10.1016/j.metabol.2009.08.001 PMC2932671 19800083

[21] Hashimoto Y Suzuki H Aiso S Niikura T Nishimoto I Matsuoka M. Involvement of tyrosine kinases and STAT3 in humanin-mediated neuroprotection. Life Sci (2005) 77:3092–104. doi: 10.1016/j.lfs.2005.03.031 16005025

[22] Jia Y Lue YH Swerdloff R Lee KW Cobb LJ Cohen P. The cytoprotective peptide humanin is induced and neutralizes bax after pro-apoptotic stress in the rat testis. Andrology (2013) 1:651–9. doi: 10.1111/j.2047-2927.2013.00091.x PMC3696635 23686888

[23] Ming W Lu G Xin S Huanyu L Yinghao J Xiaoying L. Mitochondria related peptide MOTS-c suppresses ovariectomy-induced bone loss *via* AMPK activation. Biochem Biophys Res Commun (2016) 476:412–9. doi: 10.1016/j.bbrc.2016.05.135 27237975

[24] Qin Q Jin J He F Zheng Y Li T Zhang Y. Humanin promotes mitochondrial biogenesis in pancreatic MIN6 β-cells. Biochem Biophys Res Commun (2018) 497:292–7. doi: 10.1016/j.bbrc.2018.02.071 29432738

[25] Chiba T Yamada M Hashimoto Y Sato M Sasabe J Kita Y. Development of a femtomolar-acting humanin derivative named colivelin by attaching activity-dependent neurotrophic factor to its n terminus: characterization of colivelin-mediated neuroprotection against alzheimer's disease-relevant insults *in vitro* and *in vivo* . J Neurosci (2005) 25:10252–61. doi: 10.1523/JNEUROSCI.3348-05.2005 PMC6725789 16267233

[26] Chiba T Yamada M Sasabe J Terashita K Aiso S Matsuoka M. Colivelin prolongs survival of an ALS model mouse. Biochem Biophys Res Commun (2006) 343:793–8. doi: 10.1016/j.bbrc.2006.02.184 16564029

[27] Sari Y Chiba T Yamada M Rebec GV Aiso S. A novel peptide, colivelin, prevents alcohol-induced apoptosis in fetal brain of C57BL/6 mice: signaling pathway investigations. Neuroscience (2009) 164:1653–64. doi: 10.1016/j.neuroscience.2009.09.049 PMC2783970 19782727

[28] Zhao H Feng Y Wei C Li Y Ma H Wang X. Colivelin rescues ischemic neuron and axons involving JAK/STAT3 signaling pathway. Neuroscience (2019) 416:198–206. doi: 10.1016/j.neuroscience.2019.07.020 31374230

[29] Flurkey K Currer JM Harrison DE. The mouse in aging research. In: Fox JG, editor. The mouse in biomedical research, 2nd Edition. Burlington, MA, USA: American College Laboratory Animal Medicine (Elsevier) (2007).

[30] Hubbard WJ Choudhry M Schwacha MG Kerby JD Rue LW 3rd Bland KI. Cecal ligation and puncture. Shock (2005) 24(Suppl 1):52–7. doi: 10.1097/01.shk.0000191414.94461.7e 16374373

[31] Shrum B Anantha RV Xu SX Donnelly M Haeryfar SM McCormick JK. A robust scoring system to evaluate sepsis severity in an animal model. BMC Res Notes (2014) 7:233. doi: 10.1186/1756-0500-7-233 24725742 PMC4022086

[32] Zingarelli B Coopersmith CM Drechsler S Efron P Marshall JC Moldawer L. Part I: Minimum quality threshold in preclinical sepsis studies (MQTiPSS) for study design and humane modeling endpoints. Shock (2019) 51:10–22. doi: 10.1097/SHK.0000000000001243 30106874 PMC6296871

[33] Mullane KM Kraemer R Smith B. Myeloperoxidase activity as a quantitative assessment of neutrophil infiltration into ischemic myocardium. J Pharmacol Methods (1985) 14:157–67. doi: 10.1016/0160-5402(85)90029-4 2997548

[34] Wolthuis EK Vlaar AP Choi G Roelofs JJ Juffermans NP Schultz MJ. Mechanical ventilation using non-injurious ventilation settings causes lung injury in the absence of pre-existing lung injury in healthy mice. Crit Care (2009) 13:R1. doi: 10.1186/cc7688 19152704 PMC2688111

[35] Wiesinger A Peters W Chappell D Kentrup D Reuter S Pavenstädt H. Nanomechanics of the endothelial glycocalyx in experimental sepsis. PloS One (2013) 8:e80905. doi: 10.1371/journal.pone.0080905 24278345 PMC3835794

[36] Schneider CA Rasband WS Eliceiri KW. NIH Image to ImageJ: 25 years of image analysis. Nat Methods (2012) 9:671–5. doi: 10.1038/nmeth.2089 PMC5554542 22930834

[37] Steppan J Hofer S Funke B Brenner T Henrich M Martin E. Sepsis and major abdominal surgery lead to flaking of the endothelial glycocalix. J Surg Res (2011) 165:136–41. doi: 10.1016/j.jss.2009.04.034 19560161

[38] Sallisalmi M Tenhunen J Yang R Oksala N Pettilä V. Vascular adhesion protein-1 and syndecan-1 in septic shock. Acta Anaesthesiol Scand (2012) 56:316–22. doi: 10.1111/j.1399-6576.2011.02578.x 22150439

[39] Nelson A Berkestedt I Schmidtchen A Ljunggren L Bodelsson M. Increased levels of glycosaminoglycans during septic shock: relation to mortality and the antibacterial actions of plasma. Shock (2008) 30:623–7. doi: 10.1097/SHK.0b013e3181777da3 18497712

[40] Burmeister DM Heard TC Chao T Alcover K Wagner A Chung KK. A prospective observational study comparing clinical sepsis criteria to protein biomarkers reveals a role for vascular dysfunction in burn sepsis. Crit Care Explor (2022) 4:e0610. doi: 10.1097/CCE.0000000000000610 35018348 PMC8735811

[41] Martin-Fernandez M Vaquero-Roncero LM Almansa R Gómez-Sánchez E Martín S Tamayo E. Endothelial dysfunction is an early indicator of sepsis and neutrophil degranulation of septic shock in surgical patients. BJS Open (2020) 4:524–34. doi: 10.1002/bjs5.50265 PMC7260414 32073224

[42] Giuliano JS Jr Lahni PM Harmon K Wong HR Doughty LA Carcillo JA. Admission angiopoietin levels in children with septic shock. Shock (2007) 28:650–4. PMC2754128 18092380

[43] Giuliano JS Jr Tran K Li FY Shabanova V Tala JA Bhandari V. The temporal kinetics of circulating angiopoietin levels in children with sepsis. Pediatr Crit Care Med (2014) 15:e1–8. doi: 10.1097/PCC.0b013e3182a553bb PMC3947338 24141659

[44] Hahn WO Mikacenic C Price BL Harju-Baker S Katz R Himmelfarb J. Host derived biomarkers of inflammation, apoptosis, and endothelial activation are associated with clinical outcomes in patients with bacteremia and sepsis regardless of microbial etiology. Virulence (2016) 7:387–94. doi: 10.1080/21505594.2016.1144003 PMC4871669 26818467

[45] Agouridakis P Kyriakou D Alexandrakis MG Prekates A Perisinakis K Karkavitsas N. The predictive role of serum and bronchoalveolar lavage cytokines and adhesion molecules for acute respiratory distress syndrome development and outcome. Respir Res (2002) 3:25. doi: 10.1186/rr193 12537603 PMC150513

[46] Flori HR Ware LB Glidden D Matthay MA. Early elevation of plasma soluble intercellular adhesion molecule-1 in pediatric acute lung injury identifies patients at increased risk of death and prolonged mechanical ventilation. Pediatr Crit Care Med (2003) 4:315–21. doi: 10.1097/01.PCC.0000074583.27727.8E 12831413

[47] Calfee CS Eisner MD Parsons PE Thompson BT Conner ER Jr Matthay MA. NHLBI acute respiratory distress syndrome clinical trials network. soluble intercellular adhesion molecule-1 and clinical outcomes in patients with acute lung injury. Intensive Care Med (2009) 35:248–57. doi: 10.1007/s00134-008-1235-0 PMC2790376 18670758

[48] Vassiliou AG Mastora Z Orfanos SE Jahaj E Maniatis NA Koutsoukou A. Elevated biomarkers of endothelial dysfunction/activation at ICU admission are associated with sepsis development. Cytokine (2014) 69:240–7. doi: 10.1016/j.cyto.2014.06.010 25016133

[49] Wright JK Hayford K Tran V Al Kibria GM Baqui A Manajjir A. Biomarkers of endothelial dysfunction predict sepsis mortality in young infants: a matched case-control study. BMC Pediatr (2018) 18:118. doi: 10.1186/s12887-018-1087-x 29571293 PMC5866512

[50] Yu WK McNeil JB Wickersham NE Shaver CM Bastarache JA Ware LB. Angiopoietin-2 outperforms other endothelial biomarkers associated with severe acute kidney injury in patients with severe sepsis and respiratory failure. Crit Care (2021) 25:48. doi: 10.1186/s13054-021-03474-z 33541396 PMC7859898

[51] Leite AR Borges-Canha M Cardoso R Neves JS Castro-Ferreira R Leite-Moreira A. Novel biomarkers for evaluation of endothelial dysfunction. Angiology (2020) 71:397–410. doi: 10.1177/0003319720903586 32077315

[52] Kapur NK Heffernan KS Yunis AA Parpos P Kiernan MS Sahasrabudhe NA. Usefulness of soluble endoglin as a noninvasive measure of left ventricular filling pressure in heart failure. Am J Cardiol (2010) 106:1770–6. doi: 10.1016/j.amjcard.2010.08.018 PMC3353730 21126621

[53] Yanavitski M Givertz MM. Novel biomarkers in acute heart failure. Curr Heart Fail Rep (2011) 8:206–11. doi: 10.1007/s11897-011-0065-5 21681444

[54] Walley KR Francis GA Opal SM Stein EA Russell JA Boyd JH. The central role of proprotein convertase Subtilisin/Kexin type 9 in septic pathogen lipid transport and clearance. Am J Respir Crit Care Med (2015) 192:1275–86. doi: 10.1164/rccm.201505-0876CI 26252194

[55] Ding Z Liu S Wang X Deng X Fan Y Sun C. Hemodynamic shear stress *via* ROS modulates PCSK9 expression in human vascular endothelial and smooth muscle cells and along the mouse aorta. Antioxid Redox Signal (2015) 22:760–71. doi: 10.1089/ars.2014.6054 PMC4361218 25490141

[56] Le Bras M Roquilly A Deckert V Langhi C Feuillet F Sébille V. Plasma PCSK9 is a late biomarker of severity in patients with severe trauma injury. J Clin Endocrinol Metab (2013) 98:E732–6. doi: 10.1210/jc.2012-4236 23450051

[57] Vecchié A Bonaventura A Meessen J Novelli D Minetti S Elia E. ALBIOS biomarkers study investigators. PCSK9 is associated with mortality in patients with septic shock: data from the ALBIOS study. J Intern Med (2021) 289:179–92. doi: 10.1111/joim.1315 32686253

[58] Dounousi E Tellis C Pavlakou P Duni A Liakopoulos V Mark PB. Association between PCSK9 levels and markers of inflammation, oxidative stress, and endothelial dysfunction in a population of nondialysis chronic kidney disease patients. Oxid Med Cell Longev (2021) 2021:6677012. doi: 10.1155/2021/6677012 34336112 PMC8318757

[59] Smart L Bosio E Macdonald SPJ Dull R Fatovich DM Neil C. Glycocalyx biomarker syndecan-1 is a stronger predictor of respiratory failure in patients with sepsis due to pneumonia, compared to endocan. J Crit Care (2018) 47:93–8. doi: 10.1016/j.jcrc.2018.06.015 29936329

[60] Nieuwdorp M Meuwese MC Mooij HL van Lieshout MH Hayden A Levi M. Tumor necrosis factor-alpha inhibition protects against endotoxin-induced endothelial glycocalyx perturbation. Atherosclerosis (2009) 202:296–303. doi: 10.1016/j.atherosclerosis.2008.03.024 18550063

[61] Nelson A Berkestedt I Bodelsson M. Circulating glycosaminoglycan species in septic shock. Acta Anaesthesiol Scand (2014) 58:36–43. doi: 10.1111/aas.12223 24341693

[62] Yu H Lee H Herrmann A Buettner R Jove R. Revisiting STAT3 signalling in cancer: new and unexpected biological functions. Nat Rev Cancer (2014) 14:736–46. doi: 10.1038/nrc3818 25342631

[63] Wegrzyn J Potla R Chwae YJ Sepuri NB Zhang Q Koeck T. Function of mitochondrial Stat3 in cellular respiration. Science (2009) 323(5915):793–7. doi: 10.1126/science.1164551 PMC2758306 19131594

[64] Kim SJ Guerrero N Wassef G Xiao J Mehta HH Cohen P. The mitochondrial-derived peptide humanin activates the ERK1/2, AKT, and STAT3 signaling pathways and has age-dependent signaling differences in the hippocampus. Oncotarget (2016) 7(30):46899–912. doi: 10.18632/oncotarget.10380 PMC5216912 27384491

[65] Xu S Pan X Mao L Pan H Xu W Hu Y. Phospho-Tyr705 of STAT3 is a therapeutic target for sepsis through regulating inflammation and coagulation. Cell Commun Signal (2020) 18:104. doi: 10.1186/s12964-020-00603-z 32641132 PMC7341624

[66] Williamson L Ayalon I Shen H Kaplan J. Hepatic STAT3 inhibition amplifies the inflammatory response in obese mice during sepsis. Am J Physiol Endocrinol Metab (2019) 316(2):E286–92. doi: 10.1152/ajpendo.00341.2018 PMC6397363 30576248

[67] Imbaby S Matsuda N Tomita K Hattori K Palikhe S Yokoo H. Beneficial effect of STAT3 decoy oligodeoxynucleotide transfection on organ injury and mortality in mice with cecal ligation and puncture-induced sepsis. Sci Rep (2020) 10:15316. doi: 10.1038/s41598-020-72136-x 32943679 PMC7498613

[68] Balic JJ Albargy H Luu K Kirby FJ Jayasekara WSN Mansell F. STAT3 serine phosphorylation is required for TLR4 metabolic reprogramming and IL-1β expression. Nat Commun (2020) 11:3816. doi: 10.1038/s41467-020-17669-5 32732870 PMC7393113

[69] Zhao J Yu H Liu Y Gibson SA Yan Z Xu X. Protective effect of suppressing STAT3 activity in LPS-induced acute lung injury. Am J Physiol Lung Cell Mol Physiol (2016) 311:L868–80. doi: 10.1152/ajplung.00281.2016 PMC5130536 27638904

[70] Carling D Viollet B. Beyond energy homeostasis: the expanding role of AMP-activated protein kinase in regulating metabolism. Cell Metab (2015) 21(6):799–804. doi: 10.1016/j.cmet.2015.05.005 26039446

[71] Kwon C Sun JL Jeong JH Jung TW. Humanin attenuates palmitate-induced hepatic lipid accumulation and insulin resistance *via* AMPK-mediated suppression of the mTOR pathway. Biochem Biophys Res Commun (2020) 526:539–45. doi: 10.1016/j.bbrc.2020.03.128 32245619

[72] Muzumdar RH Huffman DM Calvert JW Jha S Weinberg Y Cui L. Acute humanin therapy attenuates myocardial ischemia and reperfusion injury in mice. Arterioscler Thromb Vasc Biol (2010) 30:1940–8. doi: 10.1161/ATVBAHA.110.205997 PMC2941397 20651283

[73] Gong H Tai H Huang N Xiao P Mo C Wang X. Nrf2-SHP cascade-mediated STAT3 inactivation contributes to AMPK-driven protection against endotoxic inflammation. Front Immunol (2020) 11:414. doi: 10.3389/fimmu.2020.00414 32210977 PMC7076194

[74] Inata Y Kikuchi S Samraj RS Hake PW O'Connor M Ledford JR. Autophagy and mitochondrial biogenesis impairment contributes to age-dependent liver injury in experimental sepsis: dysregulation of AMP-activated protein kinase pathway. FASEB J (2018) 32:728–41. doi: 10.1096/fj.201700576R74 PMC5888394 28974562

[75] Inata Y Piraino G Hake PW O'Connor M Lahni P Wolfe V. Age-dependent cardiac function during experimental sepsis: effect of pharmacological activation of AMP-activated protein kinase by AICAR. Am J Physiol Heart Circ Physiol (2018) 315:H826–37. doi: 10.1152/ajpheart.00052.2018 PMC6230907 29979626

[76] Evangelou A Zikos C Benaki D Pelecanou M Bouziotis P Papadopoulos M. *In vitro* binding and *in vivo* biodistribution studies of the neuroprotective peptide humanin using [^125^I]humanin derivatives. Peptides (2009) 30:2409–17. doi: 10.1016/j.peptides.2009.07.028 19666070

