# Supplementary material for: Colivelin, a synthetic derivative of humanin, ameliorates endothelial injury and glycocalyx shedding after sepsis in mice
Source: Front Immunol. 2022 Sep 2;13:984298. doi: 10.3389/fimmu.2022.984298 (PMC9478210; doi:10.3389/fimmu.2022.984298)
Supplement: Supplementary file 1 [file DataSheet_1.pdf]

Supplementary Material

Supplementary Figures

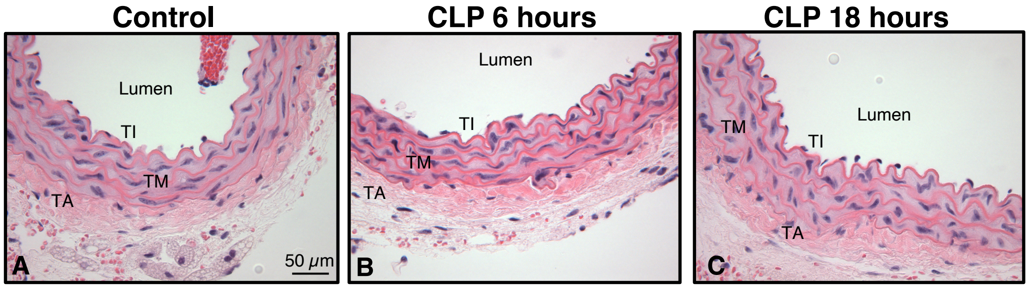

Supplementary Figure S1. Representative histology photomicrographs of thoracic aorta sections of a control mouse (**A**) or mice subjected to polymicrobial sepsis at 6 h (**B**) and 18 h (**C**) after cecal ligation and puncture (CLP) with normal morphology of tunica intima (TI), tunica media (TM), and tunica adventitia (TA). Magnification x400. A similar pattern was seen in n=4-8 different tissue sections in each experimental group.

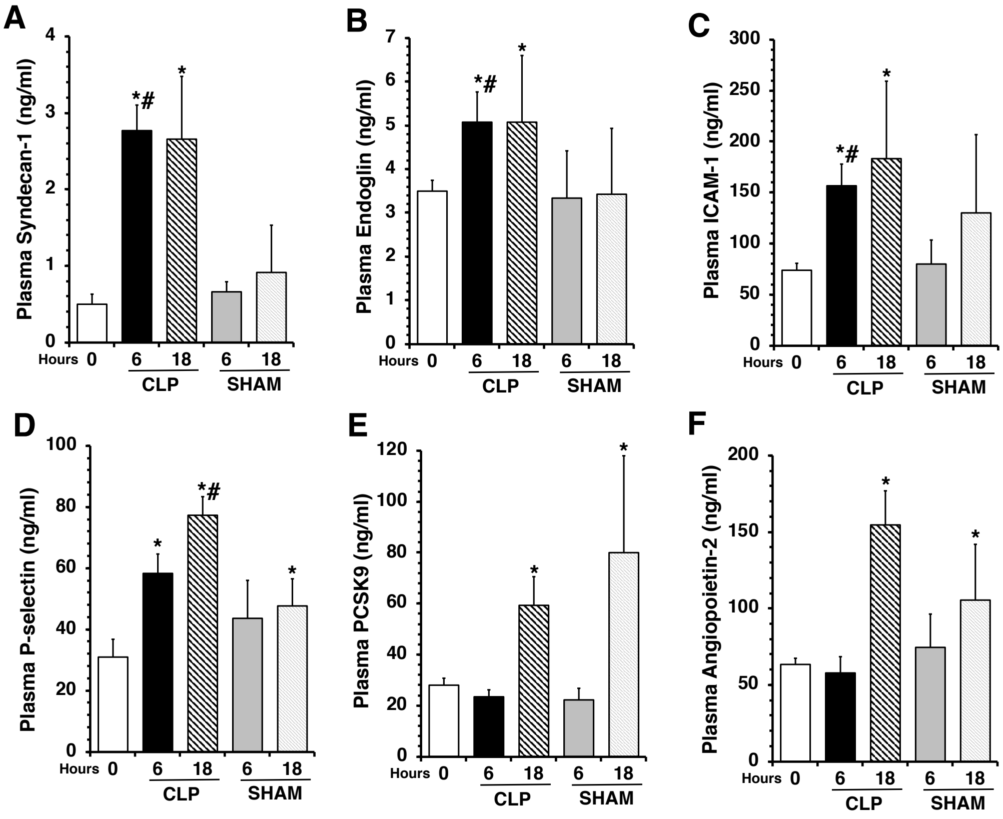

Supplementary Figure S2. Plasma levels of Syndecan-1 (**A**), Endoglin (**B**), ICAM-1 (**C**), P-selectin (**D**), PCSK9 (**E**), and Angiopoietin-2 (**F**) at 0 h, 6 h and 18 h after cecal ligation and puncture (CLP)

or sham surgery (SHAM). Data represents the mean ± SEM of 10-13 mice for group (n=10 control group at 0 h, n=13 at 6 h CLP, n=11 at 18 h CLP, n=9 at 6 h SHAM, n=8 at 18 h SHAM). *Represents $P < 0.05$ *versus* control mice at time 0; #represents $P < 0.05$ *versus* sham mice.

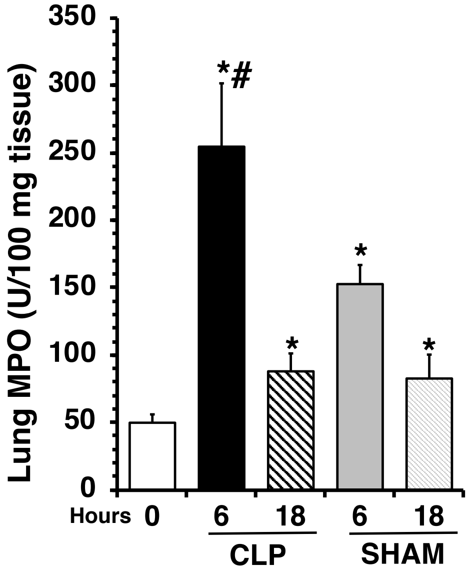

Supplementary Figure S3. Activity of myeloperoxidase (MPO) in lung (**A**), liver (**B**), kidney (**C**) at 6 h after cecal ligation and puncture (CLP) or sham surgery (SHAM). Data represents the mean ± SEM of 8-17 mice for group (n=17 control group at 0 h, n=13 at 6 h CLP, n=11 at 18 h CLP, n=9 at 6 h SHAM, n=8 at 18 h SHAM). *Represents $P < 0.05$ *versus* control mice at time 0; #represents $P < 0.05$ *versus* sham mice.
